# Age-induced prostaglandin E_2_ impairs mitochondrial fitness and increases mortality to influenza infection

**DOI:** 10.1038/s41467-022-34593-y

**Published:** 2022-11-09

**Authors:** Judy Chen, Jane C. Deng, Rachel L. Zemans, Karim Bahmed, Beata Kosmider, Min Zhang, Marc Peters-Golden, Daniel R. Goldstein

**Affiliations:** 1 Department of Internal Medicine, University of Michigan, Ann Arbor, MI 48109 USA; 2 Program in Immunology, University of Michigan, Ann Arbor, MI 48109 USA; 3 Division of Pulmonary and Critical Care Medicine, University of Michigan, Ann Arbor, MI 48109 USA; 4 Center for Inflammation and Lung Research, Department of Microbiology, Immunology, and Inflammation, Temple University, Philadelphia, PA 19140 USA; 5 Department of Thoracic Medicine and Surgery, Temple University, Philadelphia, PA 19140 USA; 6 Department of Biostatistics, University of Michigan, Ann Arbor, MI 48109 USA; 7 Department of Microbiology and Immunology, University of Michigan, Ann Arbor, MI 48109 USA

**Keywords:** Infection, Ageing, Influenza virus

## Abstract

Aging impairs the immune responses to influenza A virus (IAV), resulting in increased mortality to IAV infections in older adults. However, the factors within the aged lung that compromise host defense to IAV remain unknown. Using a murine model and human samples, we identified prostaglandin E_2_ (PGE_2_), as such a factor. Senescent type II alveolar epithelial cells (AECs) are overproducers of PGE_2_ within the aged lung. PGE_2_ impairs the proliferation of alveolar macrophages (AMs), critical cells for defense against respiratory pathogens, via reduction of oxidative phosphorylation and mitophagy. Importantly, blockade of the PGE_2_ receptor EP2 in aged mice improves AM mitochondrial function, increases AM numbers and enhances survival to IAV infection. In conclusion, our study reveals a key mechanism that compromises host defense to IAV, and possibly other respiratory infections, with aging and suggests potential new therapeutic or preventative avenues to protect against viral respiratory disease in older adults.

## Abstract (web summary)

Ageing has been associated with impaired function of alveolar macrophages and increased susceptibility, mortality and complications as a result of viral infection. Here, the authors show that prostaglandin E_2_ is one of the factors responsible for impairing immune defences against influenza during ageing.

## Introduction

Influenza A virus (IAV) remains a serious health burden that disproportionately affects older individuals. Most young people who contract IAV only experience mild to moderate symptoms, but older individuals (≥65 years of age) experience higher rates of susceptibility, mortality, and complications such as secondary bacterial infections^1,2^. For example, 86% of the deaths due to 2017–2018 seasonal IAV infections in the United States (US) occurred in individuals 65 years or older^3^, even though this age group is only 13% of the total US population^4^. Compounding this issue, the three classes of anti-IAV medications: adamantanes, neuraminidase inhibitors, and cap-dependent endonuclease inhibitors, exhibit limited effectiveness in older individuals^5,6^. Overall, the susceptibility of older individuals to IAV is of increasing concern as the average age of the world’s population continues to rise across the globe^7^.

Alveolar macrophages (AMs) are airway and airspace resident macrophages that maintain lung homeostasis and respond rapidly to respiratory pathogens. The importance of AMs in controlling IAV infection is exhibited by the rapid weight loss, increased tissue damage, and poor survival in IAV-infected murine models of genetic AM deficiency or pharmacological AM depletion^8,9^. Adoptive transfer experiments in which AMs isolated from young mice are transferred into aged mice and vice versa show that the aging-associated transcriptomic differences in AMs before infection are driven by the local aged lung microenvironment^10^. However, the specific factors within the aged lung that contribute to the aging-associated defects of AMs remain unknown, thereby precluding our ability to target the underlying signals.

Prostaglandin E_2_ (PGE_2_) is a immune-modulating lipid and prior studies, including our own, have found that PGE_2_ levels are increased in the lung with aging in mice^11,12^. However, it is not clear if aging elevates PGE_2_ levels human lungs. PGE_2_ is associated with various inflammatory conditions including rheumatoid arthritis^13^ and cancer^14^, as well as infections including IAV infection^15^. However, the key clinically relevant question of whether PGE_2_ is a causal factor in the aged lung contributing to compromised host defense to viral infection remains unanswered.

Cellular senescence is a hallmark of aging^16^ that is characterized by the senescence-associated secretory phenotype (SASP), in which senescent cells secrete inflammatory mediators such as cytokines, chemokines, and growth factors, at steady state^17,18^. As senescent cells accumulate with age, increased production of SASP factors contribute to the low-grade, chronic inflammation associated with aging^19,20^. Although the majority of known SASP factors are proteins, emerging evidence indicates that lipids also contribute to SASP^21,22^. However, it remains unclear whether senescence within the lung contributes to elevated PGE_2_ levels with aging. Moreover, the identity of cell type(s) that contribute to age-elevated PGE_2_ either independently or in response to viral infection are unknown.

Here, we tested our hypothesis of whether age-elevated PGE_2_ is a critical factor that impairs host defense to IAV infection. We show that senescent type II alveolar epithelial cells (AECs) are a major contributor to age-associated elevations of PGE_2_ in the lung before and after IAV infection in mice. We also show that PGE_2_ increases in the lung with age in humans. We reveal that PGE_2_, through the EP2 receptor, limits AM proliferation and mitochondrial fitness with aging. Critically, we show that blocking the EP2 receptor improves survival of aged, but not young, mice to lethal IAV infection. Our study has revealed a novel pathophysiological cross talk between type II AECs and AMs via elevated PGE_2_ that impairs host defense to IAV with aging.

## Results

### Type II AECs are a primary source of PGE_2_ in the aged lung

Prior studies, including our own, have shown that PGE_2_ is increased in the aged lung, specifically within the bronchoalveolar lavage fluid (BALF), in non-infected mice^11,12^. However, cellular sources driving the age-associated increase of PGE_2_ remain unknown. To identify which lung cell type(s) secrete(s) PGE_2_ with aging, we initially utilized a scRNA-seq dataset (Tabula Muris, GEO GSE109774)^23^ to identify the cell types that highly express the enzymes COX1 and COX2, both critical, rate-limiting enzymes of PGE_2_ production^24,25^. COX1 is encoded by the gene *Ptgs1* and COX2 is encoded by the gene *Ptgs2*. Our initial query showed that epithelial cells exhibited high expression of both genes (Supplementary Fig. 1a). To determine if type I or type II alveolar epithelial cells (AECs) are potent PGE_2_ producers, we re-analyzed publicly available scRNA-seq data of AECs (GEO GSE113049) and found that type II AECs express ~5.5-fold higher transcript levels of *Ptgs1* and ~8.5-fold higher transcript levels of *Ptgs2*, relative to type I AECs (Supplementary Fig. 1b). Therefore, we considered type II, rather than type I, AECs as potential contributors to PGE_2_ levels with aging. Given that myeloid immune cells are known secretors of PGE_2_^26–28^_,_ we also considered that AMs, lung resident myeloid cells, as candidate contributors to the overproduction of PGE_2_ with aging. The localization of both type II AECs and AMs at the alveolar surface make them prime candidates to account for the excess PGE_2_ levels found in the BALF of aged mice.

**Fig. 1:**
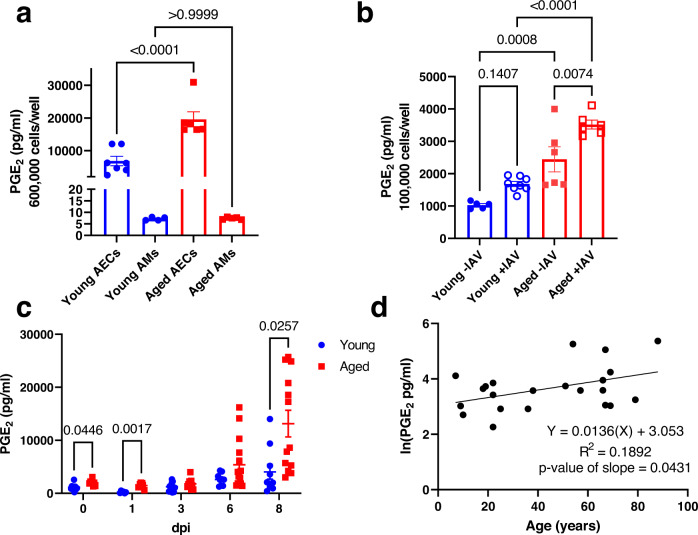
Type II AECs are a primary source of PGE_2_ in the aged lung. **a**, **b** Primary type II AECs and AMs were isolated from young and aged C57BL/6 mice and then cultured ex vivo. Cell culture medium was collected after 2 days and analyzed by ELISA for PGE_2_. **a** Basal PGE_2_ secretion by primary type II AECs and AMs in culture. *n* = 7 for Young AECs, *n* = 4 for young AMs, *n* = 6 for Aged AECs, *n* = 5 for Aged AMs. **b** Primary type II AECs were isolated from young and aged C57BL/6 mice and were infected ex vivo with PR8 IAV (MOI = 0.1). Cell culture medium was collected following 2 days of culture and analyzed for PGE_2_ via ELISA. *n* = 5 for Young -IAV, *n* = 8 for Young +IAV, *n* = 6 for Aged -IAV, *n* = 6 for Aged +IAV. **c** PGE_2_ from the BALF of young (2–4 months) and aged (18–22 months) female C57BL/6 mice infected with 400 pfu of PR8 IAV intranasally measured at 0, 1, 3, 6 and 8 days post infection (dpi). *n* = 6 for young 1 dpi. *n* = 7 for aged 1 dpi. *n* = 8 for aged 1 dpi. *n* = 9 for young 0 dpi and young 6 dpi. *n* = 10 for young 8 dpi. *n* = 11 for aged 3 dpi. *n* = 12 for young 3 dpi. *n* = 13 for aged 8 dpi. *n* = 15 for aged 6 dpi. For panels **a**–**c**, statistical significance analyzed by ANOVA with Tukey post hoc test, error bars represent SEM and each point represents a biological replicate. **d** PGE_2_ levels were measured from the BALF of 22 healthy human subjects. The correlation between ln-normalized PGE_2_ levels and age was calculated using simple linear regression. The *p* value of the slope was calculated based on the null hypothesis of slope = 0 and the alternative hypothesis of slope ≠ 0. Each point represents one biological replicate. Source data are provided as a Source Data file.

We isolated and cultured both AMs and type II AECs from non-infected young (2–4 months of age) and aged (18–22 months of age) female C57BL/6 mice. Following 2 days of culture, we collected the cell culture medium and measured PGE_2_ by ELISA. We found that type II AECs produced PGE_2_ at 3 orders of magnitude higher than AMs regardless of host age (Fig. 1a). Importantly, type II AECs from aged mice produced ~2-fold more PGE_2_ than type II AECs from young mice (Fig. 1a). Type II AECs isolated from young and aged mice had similar rates of viability and apoptosis following cell culture (Supplementary Fig. 1c, d), indicating that the differences in PGE_2_ levels are not due to culture viability differences. These results suggest that type II AECs, but not AMs, are a major source of PGE_2_ in the BALF at steady state, and aging increases the production of PGE_2_ by type II AECs.

We next determined if IAV infection increases PGE_2_ production by type II AECs and whether aging exacerbates the phenotype. Hence, we cultured type II AECs isolated from either young or aged mice, infected the AECs ex vivo with IAV at a multiplicity of infection (MOI) of 0.1 for 2 days and subsequently measured PGE_2_ in the culture medium via ELISA. Importantly, IAV led to increased PGE_2_ production by ~1.5-fold in aged type II AECs, and infected type II AECs from aged mice producing ~2-fold higher levels of PGE_2_ as compared to infected type II AECs isolated from young mice (Fig. 1b).

We next examined how aging and IAV infection impacts PGE_2_ levels within the BALF in vivo. Hence, we infected young and aged female C57BL/6 mice with 400 plaque forming units (pfu) of H1N1 A/PR/8/34 IAV intranasally (i.n.) as 400 pfu is the lethal dose 70% (LD_70_) in aged mice (Supplementary Fig. 1e, survival tracked up to 15 days post infection (dpi)) and collected the BALF at 0, 1, 3, 6, and 8 dpi for the PGE_2_ ELISA. Prior to infection (i.e., 0 dpi), aged mice exhibited ~2-fold higher levels of PGE_2_ as compared to young mice (Fig. 1c) and at 8 dpi aged mice exhibited a ~3-fold increase of PGE_2_ in the BALF as compared with infected young mice (Fig. 1c). Overall, these ex vivo and in vivo results demonstrate that aging upregulates PGE_2_ secretion within the lung both in the presence and absence of IAV infection.

We next determined whether the age-enhanced levels of PGE_2_ in the BALF is a genotype- or sex- specific phenomenon, by measuring PGE_2_ in the BALF of young (6 months) and aged (22 months) male non-infected UM-HET3 mice, a 4-way crossed outbred mouse strain used by the National Institute on Aging Interventions Testing Program^29^. Similar to the female C57BL/6 mice, the aged male UM-HET3 exhibited a ~3-fold increase of PGE_2_ within the BALF compared to the young mice (Supplementary Fig. 1f).

To determine if BALF PGE_2_ levels increase with aging in humans, we analyzed PGE_2_ levels in BALF collected from 22 healthy human patients (Supplementary Table 1). Using simple linear regression, we found a positive correlation between age and PGE_2_ levels in the BALF (Fig. 1d). Taken together, these results indicate that increasing PGE_2_ levels in the BALF with aging is a conserved finding across sex, genotype, and species.

### Senescence increases PGE_2_ by type II AECs

**Fig. 2:**
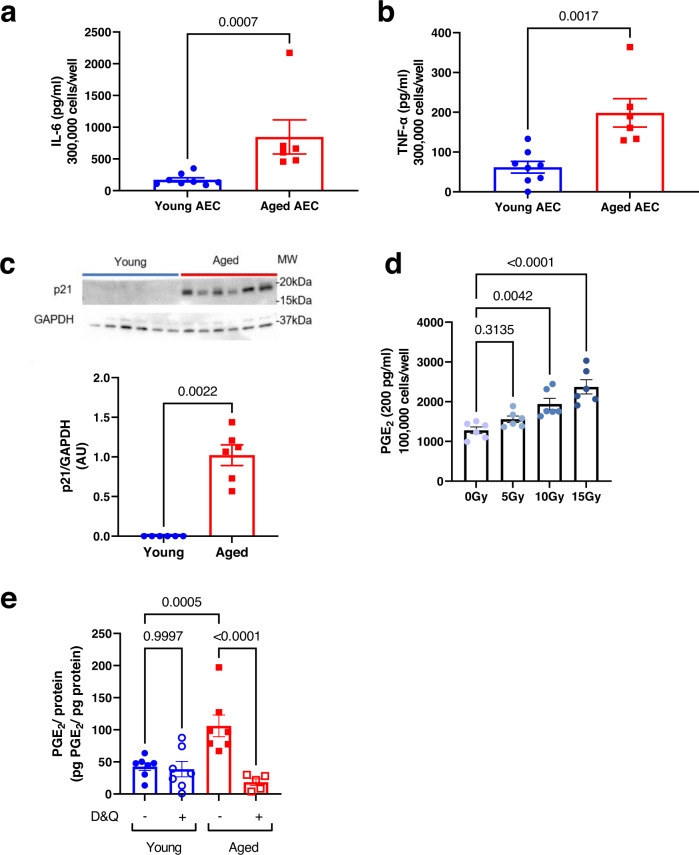
Senescence increases PGE_2_ by type II AECs. **a**, **b** Primary type II AECs and AMs were isolated from young and aged C57BL/6 mice and then cultured ex vivo. Cell culture medium was collected after 2 days and analyzed by ELISA for secreted factors. **a** IL-6 secreted by primary type II AECs in culture. **b** TNF-α secreted by primary type II AECs in culture. *n* = 8 for young AEC and *n* = 6 for Aged AEC. **c** Primary type II AECs isolated from young and aged C57BL/6 mice were lysed and analyzed for p21 expression by Western blot. *n* = 6/group. For panels **a**–**c**, statistical significance analyzed by Mann–Whitney, error bars represents SEM and each point represents a biological replicate. **d** Primary type II AECs isolated from young C57BL/6 mice were irradiated with increasing levels of radiation (0, 5, 10, or 15 Gy) to induce senescence. Cell culture medium was collected and analyzed for PGE_2_ levels by ELISA. *n* = 6/group. **e** Primary type II AECs isolated from young and aged C57BL/6 mice were treated with D&Q (20 nM/5 µM) for 48 h. Cell culture medium was collected and analyzed for PGE_2_ by ELISA. PGE_2_ levels were normalized to the cellular protein levels of adherent, live type II AECs. *n* = 7 for Young control, Young D&Q, and Aged control. *n* = 5 for Aged D&Q. For panels **d** and **e,** statistical significance analyzed by ANOVA with Tukey post hoc test, error bars represent SEM and each point represents a biological replicate. Source data are provided as a Source Data file.

Next, we sought to determine a cellular mechanism by which aging increases PGE_2_ production by type II AECs, and focused our attention on senescence as a possible mechanism. To determine if senescence increases PGE_2_ secretion by type II AECs and if PGE_2_ is a SASP factor within the lung, we first characterized whether type II AECs from aged mice exhibit features of senescence. We found that type II AECs from aged mice exhibited a ~2–3-fold increased secretion of two SASP factors, IL-6 and TNF-α, as compared to type II AECs from non-infected young mice (Fig. 2a, b). Type II AECs from young mice had undetectable levels of the senescence marker p21, whereas type II AECs from aged mice exhibited high expression of p21 (Fig. 2c). Overall, these results show that type II AECs isolated from aged mice exhibit evidence of senescence.

To determine if senescence increases PGE_2_ secretion, we irradiated type II AECs isolated from young mice with either 0, 5, 10, or 15 Gy of radiation to induce a senescent phenotype within the cells^30^. The levels of p21 in type II AECs increased with increasing doses of radiation, confirming the induction of a senescence phenotype (Supplementary Fig. 2a). We then measured PGE_2_ in the cell culture medium of the radiated type II AECs by ELISA and found increasing PGE_2_ production with increasing irradiation, correlating with p21 expression (Fig. 2d and Supplementary Fig. 2b).

To determine the necessity of senescence for PGE_2_ overproduction in type II AECs with aging, we cultured type II AECs from young and aged mice with dasatinib and quercetin (D&Q), a combination senolytic treatment that depletes senescent cells^31,32^. D&Q treatment reduced p21 expression in type II AECs from aged mice as measured by western blotting, validating our approach (Supplementary Fig. 2c). We then measured secreted PGE_2_ levels in the culture medium and normalized the results based on total protein levels of live, adherent type II AECs. The senolytic D&Q treatment reduced PGE_2_ secretion of type II AECs from aged mice by ~5.5-fold (Fig. 2e). However, the D&Q treatment did not significantly alter PGE_2_ secretion of type II AECs from young mice, as expected.

Taken together, these results suggest that PGE_2_ is a SASP factor produced by senescent type II AECs, and that senescent type II AECs are a major producer of PGE_2_ in the lung with aging.

### Blocking PGE_2_ signaling via the EP2 receptor increases AM numbers in aged mice

We next examined the in vivo consequences of age-elevated PGE_2_ levels in the lung. Of the four PGE_2_ receptors, EP2 and EP4 have been shown to be upregulated on AMs during IAV infection^15^. In particular, PGE_2_ signaling through the EP2 receptor regulates AM functions such as phagocytosis of bacteria^33^, toll-like receptor expression^34^, and production of suppressor of cytokine signaling 3 (SOCS3)^35^ and in vitro proliferation capacity^11^. Based on this and our results thus far, we hypothesized that age-elevated PGE_2_ levels reduce AM numbers and function in a way that primes aged animals to IAV infection. It has been previously shown that aging reduces the number of AMs in female C57BL/6 mice^8^, and we first confirmed that this phenotype is generalizable to young and aged male UM-HET3 mice (Supplementary Fig. 3a).

**Fig. 3:**
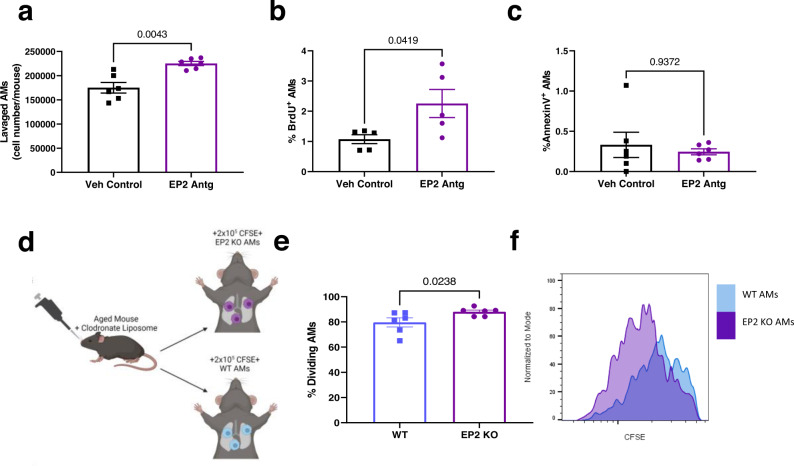
Blocking PGE_2_ signaling via the EP2 receptor increases AM numbers in aged mice. **a**–**c** Aged (18–22 months) C57BL/6 mice were given 7 daily i.p. injections of an EP2 antagonist. Concurrently, BrdU (0.8 mg/mL) was given in the drinking water ad libitum. AMs (i.e., CD45^+^ CD11c^+^ SiglecF^+^) were then collected from the BALF and analyzed by flow cytometry for BrdU incorporation, indicating proliferation, and Annexin V staining, indicating apoptosis. **a** Total cell count of lavaged AMs. *n* = 6/group. **b** Percentage of AMs that are BrdU^+^. *n* = 5/group. **c** Percentage of AMs that are Annexin V^+^. *n* = 6/group. **d**–**f** Aged mice were given clodronate-loaded liposomes to deplete resident AMs prior to an adoptive transfer of CFSE^+^ WT or EP2 KO AMs as depicted in (**d**). **e**, **f** AMs were analyzed by flow cytometry 4 weeks following transfer for CFSE dilution, a marker of proliferation *n* = 6/group. **f** Representative histogram of CFSE MFI. For panels **a**–**e**, statistical significance analyzed by Mann–Whitney, error bars represent SEM and each point represents a biological replicate. Source data are provided as a Source Data file.

We next investigated whether PGE_2_ signaling affects AM numbers in vivo, by blocking PGE_2_ signaling via intraperitoneal (i.p.) injections of an antagonist against the EP2 receptor^36^ daily for 7 days, followed by the enumeration of AMs (defined as CD45^+^ CD11c^+^ SiglecF^+^) from the BALF by flow cytometry. This treatment reduced PGE_2_-induced cAMP, a downstream molecule of the PGE_2_ receptors^14^, in the lungs (Supplementary Fig. 3b), validating our approach. Blocking PGE_2_ signaling through the EP2 receptor significantly increased total AM numbers by ~1.3-fold in aged mice (Fig. 3a). In contrast, blocking the EP2 receptor in young C57BL/6 mice did not alter total AM numbers (Supplementary Fig. 3c). These results show that excessive PGE_2_-EP2 signaling is a major factor that limits AM numbers in the aging lung.

Increased proliferation and/or reduced apoptosis could explain the increase in AM numbers seen with the EP2 receptor blockade. Using BrdU to label proliferating cells^37^, we found that blocking the EP2 receptor in aged mice led to a ~2-fold increase in the percent of BrdU-positive AMs compared to the vehicle control (Fig. 3b). On the other hand, Annexin V staining for apoptotic cells revealed that EP2 receptor blockade does not affect AM apoptosis in aged mice (Fig. 3c).

To determine if the EP2 antagonist, which was given systemically, increased AM proliferation in an AM-dependent manner, we gave aged mice clodronate-loaded liposomes i.n. to deplete resident AMs and then performed an adoptive transfer of CSFE-labeled C57BL/6 WT AMs or AMs deficient of the EP2 receptor (EP2 KO) (Fig. 3d). Four weeks following the transfer, we analyzed the proliferation of the transferred AMs as measured by CFSE dilution. We observed that the EP2 KO AMs had higher rates of proliferation compared to the WT AMs within aged mice (Fig. 3e, f). Overall, these results show that PGE_2_ limits AM numbers with aging, via the EP2 receptor, by reducing AM proliferation and without altering apoptosis.

To determine if aging alters the expression of the EP2 receptor on AMs, we re-analyzed a publicly available RNA-seq dataset (GEO GSE134397) of sorted AMs from young and aged mice^10^. The trimmed mean of M-values (TMM) normalized counts from the RNA-seq dataset showed no age-associated differences in expression of the EP2 receptors on AMs on a transcriptomic level (Supplementary Fig. 3d), indicating that the differential response to EP2 antagonism in young versus aged mice is independent of altered expression of the EP2 receptor and is instead likely to reflect the higher levels of PGE_2_.

### PGE_2_ signaling impairs the mitochondrial fitness of AMs

**Fig. 4:**
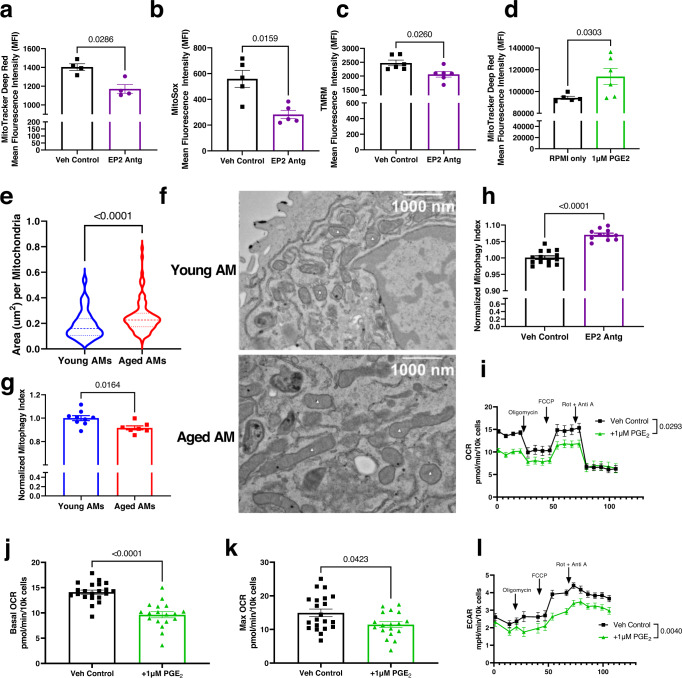
PGE_2_ signaling impairs the mitochondrial fitness of AMs. **a**–**c** Aged C57BL/6 mice were given EP2 antagonist or vehicle daily for 7 days. AMs were collected from the BALF, stained with **a** MitoTracker DeepRed to measure mitochondrial mass *n* = 4/group, **b** MitoSox to measure mitochondrial ROS *n* = 5/group, or **c** TMRM to measure mitochondrial membrane potential *n* = 6/group. The mean fluorescence intensity (MFI) was quantified. **d** AMs from young mice were cultured with 1 µM of PGE_2_ or vehicle. Cells were stained with MitoTracker DeepRed and analyzed by flow cytometry. *n* = 5 for RPMI only and *n* = 6 for 1 µM PGE_2_. For panels **a**–**d**, statistical significance analyzed by Mann–Whitney, error bars represent SEM and each point represents a biological replicate. **e**, **f** AMs were collected from the BALF of young and aged C57BL/6 mice and imaged. **e** Area of the mitochondria. Statistical significance analyzed by Mann–Whitney. *n* = 100 mitochondria/group. From top to bottom, the dashed lines in the violin plots represent upper quartile, median, and lower quartile. **f** Representative TEM images. Mitochondria indicated with small white triangles. **g**, **h** AMs from MitoQC mice were analyzed by flow cytometry. Mitophagy index was calculated based on the ratio between the mCherry and GFP signals. Mitophagy indexes were normalized within experiments to the control (i.e., young mice or vehicle). **g** AMs from young (2–3 months) and aged (22–25 months) non-infected MitoQC mice. *n* = 9 for Young AMs and *n* = 7 for Aged AMs. **h** AMs from non-infected MitoQC (9–10 months) mice, given daily injections of either EP2 antagonist or vehicle for 7 days. *n* = 14 for Veh Control and *n* = 11 for EP2 Antg. **i**–**l** AMs were cultured for 24 h with 1 µM PGE_2_ or vehicle. OCR and ECAR were analyzed by a Seahorse xFe96 analyzer. **i** OCR measurements. **j** Basal OCR measurements. **k** Maximal OCR measurements. **l** ECAR measurements. For panels **i**–**l**, *n* = 21 for Veh Control and *n* = 18 for 1 µM PGE_2_. For panels **g**, **j**, **k**, statistical significance analyzed by Mann–Whitney, error bars represent SEM and each point represents one biological replicate. For panels **i** and **l**, statistical significance analyzed by ANOVA with Tukey post hoc test and error bars represent SEM. Source data are provided as a Source Data file.

Reduced mitochondrial function, specifically the inhibition of the electron transport chain and oxidative phosphorylation, has been shown to limit proliferation in a variety of cell types such as intestinal stem cells^38^, Jurkat cells^39^, vascular smooth muscle cells^40^, and human colon cancer cells HCT116^41^. In addition, PGE_2_ has been shown to limit oxidative phosphorylation in human monocyte-derived macrophages^27^. Hence, we hypothesized that a plausible mechanism underlying the effect of PGE_2_ on AM proliferation might be reduction of mitochondrial fitness and energetic output. To test this, we collected AMs from EP2 antagonist or vehicle-treated aged mice and measured their mitochondrial mass via MitoTracker, mitochondrial reactive oxygen species (ROS) via MitoSOX, and mitochondrial membrane potential via tetramethylrhodamine methyl ester (TMRM) staining. Our results indicate that blocking the EP2 receptor reduced mitochondrial mass (Fig. 4a), mitochondrial ROS (Fig. 4b), and mitochondrial membrane potential (Fig. 4c) of AMs in aged mice. Similarly, in ex vivo culture of AMs isolated from young C57BL/6 mice, PGE_2_ treatment increased MitoTracker staining for mitochondrial mass by ~1.3-fold (Fig. 4d). Analyzing the mitochondria of AMs isolated from young and aged mice by transmission electron microscopy (TEM) also showed that the area per mitochondria in AMs also increases with age (Fig. 4e, f).

A possible avenue by which PGE_2_ could increase mitochondrial mass of AMs is by decreased mitophagy, leading to the accumulation of damaged mitochondria. Mitophagy is the autophagic recycling of damaged mitochondria^42^, and is known to be dysregulated with aging^43^. However, how aging affects mitophagy in AMs is unknown. To determine if aging alters mitophagy of AMs, we utilized a mitophagy reporter mouse model, known as the MitoQC mice. MitoQC mice express a pH-sensitive GFP and mCherry mitochondrial marker. Under neutral pH, both the mCherry and GFP fluoresce, but within the acidic environment of the autolysosome the GFP fluorescence is quenched, allowing for the in vivo detection of mitophagy at a single-cell level^44^. We analyzed AMs obtained from young (2–3 months) and aged (22–25 months) C57BL/6 MitoQC mice by flow cytometry. The mitophagy index was then calculated based on the mean fluorescence intensity (MFI) of mCherry divided by the MFI of GFP, and the results normalized to control conditions (i.e., AMs from young mice, or AMs from vehicle control treated mice). We found that AMs from aged MitoQC mice exhibited a reduced mitophagy index compared to AMs from young MitoQC mice (Fig. 4g). To determine if PGE_2_ is an age-associated factor that limits mitophagy in AMs, we treated middle-aged (i.e., 9–10 months) non-infected MitoQC mice with seven daily doses of the EP2 antagonist to block PGE_2_ signaling and detected increased mitophagy in AMs in vivo (Fig. 4h). Overall, these results indicate that aging, via elevated PGE_2_, restricts mitophagy in AMs, which could potentially lead to an accumulation of damaged mitochondria, hence increased mitochondrial mass.

The accumulation of damaged mitochondria could lead to dysregulated cellular metabolism and impaired mitochondrial oxidative phosphorylation has been shown to limit proliferation in a variety of cell types^38–41^. To understand how PGE_2_ affects cellular metabolism in AMs, we measured the oxygen consumption rate (OCR), a readout of oxidative phosphorylation, and extracellular acidification rate (ECAR), a readout of glycolysis, in AMs isolated from young mice and cultured with PGE_2_ via a Seahorse assay (Agilent)^45^. The addition of PGE_2_ reduced the OCR over the course of the assay (Fig. 4i), including reductions in both the basal OCR (Fig. 4j) and the maximal OCR (Fig. 4k). In addition, PGE_2_ also restricts the ECAR of AMs (Fig. 4l). Overall, these results suggest that PGE_2_ restricts both oxidative phosphorylation and glycolysis of AMs, to impair mitochondrial homeostasis and energy generation in AMs, and ultimately reduce AM proliferation.

We also employed an immortalized murine AM cell line, MH-S^46,47^ which showed similar mitochondrial changes as primary AMs in response to PGE_2_. We first noted that MH-S cells increased mitochondrial ROS (Supplementary Fig. 4a, b), increased mitochondrial membrane potential (Supplementary Fig. 4c, d), and reduced proliferation when cultured with PGE_2_ (Supplementary Fig. 4e). These findings are compatible with our in vivo results in which EP2 antagonism decreased mitochondrial ROS, decreased mitochondrial membrane potential in AMs (Fig. 4b, c), and increased AM cell numbers (Fig. 3a). We then employed the MH-S cell line in the Seahorse assay, which revealed that PGE_2_ lowered OCR and ECAR of MH-S cells, which is similar to our findings with the primary AMs (Supplementary Fig. 4f–i).

### Prophylactic blockade of PGE_2_ signaling through the EP2 receptor improves survival to IAV infection in aged mice

**Fig. 5:**
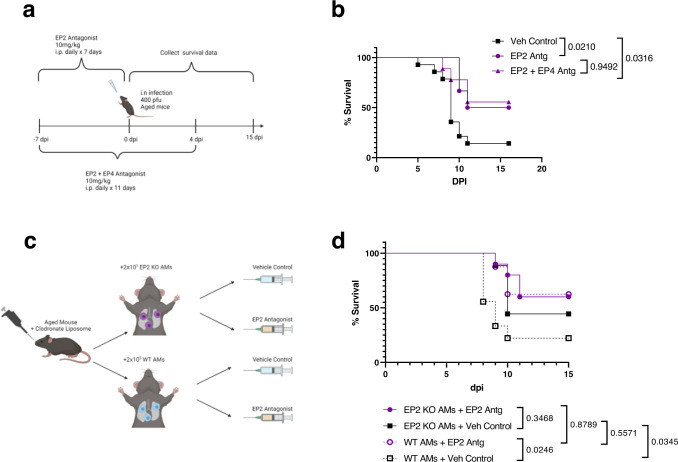
Prophylactic blockade of PGE_2_ signaling through the EP2 receptor improves survival to IAV infection in aged mice. **a**, **b** Aged (18–22 months) female C57BL/6 mice were given 7 daily i.p. injections of the EP2 antagonist starting 7 days before infection or 11 daily i.p. injections of the EP2 and EP4 antagonists starting 7 days before infection. The mice were then infected with 400 pfu of PR8 IAV intranasally as depicted in (**a**). Survival of mice (**b**) was tracked daily. *n* = 14 for Veh Control, *n* = 6 for EP2 Antg, and *n* = 9 for EP2 + EP4 Antg. **c**, **d** Aged mice were given clodronate-loaded liposomes followed by an adoptive transfer of WT or EP2 KO AMs. 4 weeks following the adoptive transfer, the mice were given EP2 antagonist or vehicle control for 7 days prior to infection with 400 pfu of PR8 IAV intranasally as depicted in (**c**). Survival of mice (**d**) was tracked daily. *n* = 8 for WT AMs+EP2 Antg. *n* = 9 for EP2 KO AMs+ Veh Control and WT AMs + Veh Control. *n* = 10 for EP2 KO AMs+ EP2 Antg. For panels **b** and **d**, survival differences were statistically determined by two-tailed Gehan–Breslow–Wilcoxon test. Schematics created in BioRender. Source data are provided as a Source Data file.

AMs are crucial for host defense against respiratory viruses such as IAV^8,9^. Our results thus far suggest that PGE_2_ limits AM numbers in non-infected aged mice. Therefore, we hypothesized that blocking PGE_2_ signaling using a 7-day prophylactic course of the EP2 antagonist in aged mice prior to infection, thereby boosting AM numbers (Fig. 3a), would improve survival to lethal IAV infection (Fig. 5a). Indeed, we found that prophylactic EP2 antagonist treatment significantly increased survival in aged IAV-infected mice from ~15% (vehicle control) to ~50% (EP2 antagonist) (Fig. 5b, survival tracked up to 16 dpi).

IAV infection upregulates both the EP2 and EP4 receptors on AMs^15^. However, a 7-day prophylactic blockade of the EP4 receptor, unlike the 7-day prophylactic blockade of the EP2 receptor, did not increase survival in aged mice infected with IAV (Supplementary Fig. 5a, b, survival tracked up to 15 dpi). To investigate potential synergy between the EP2 and EP4 receptor blockades, we administered both the EP2 and EP4 receptor antagonists prophylactically from 7 days pre-infection and to 4 dpi (days −7 to +4) to aged mice and infected the mice intranasally with IAV (Fig. 5a). Under this dosing regimen, aged mice treated with the dual EP2/EP4 antagonists exhibited a significant increase in survival versus those treated with vehicle control; however, there were no survival differences between the aged mice given the EP2 antagonist treatment alone versus the dual EP2/EP4 receptor antagonists (Fig. 5b). Thus, dual blockade treatment does not provide additional therapeutic advantages compared to the single EP2 blockade treatment.

To investigate if the EP2 antagonism improved aged mice survival to IAV infection in an AM-dependent manner, we employed an AM adoptive transfer model in which aged mice, following clodronate-liposome depletion of resident AMs, were given either WT or EP2 KO AMs. Following AM transfer, the mice were given either the EP2 antagonist or vehicle control prior to lethal IAV infection (Fig. 5c). The EP2 antagonism did not improve survival in aged mice transferred with EP2 KO AMs compared to those given vehicle control (Fig. 5d). However, EP2 antagonism improved survival in aged mice transferred with WT AMs, suggesting that the EP2 antagonism improves survival in an AM-expressed EP2-dependent manner (Fig. 5d). In addition, vehicle-treated aged mice given EP2 KO AMs showed a 2-fold increased survival compared to vehicle-treated aged mice given WT AMs, again indicating that the lack of PGE_2_ signaling through the EP2 receptor on AMs is sufficient to improve aged mice survival to IAV (Fig. 5d)

In contrast to aged mice, young C57BL/6 mice treated prophylactically with the EP2/EP4 antagonist (Supplementary Fig. 3c) and infected with a lethal dose of IAV (i.e., 800 pfu, doubled the dose given to aged mice) failed to exhibit improved survival (Supplementary Fig. 5d, survival tracked up to 13 dpi). These results are compatible with our observation that EP2 blockade failed to increase AM numbers in non-infected young mice (Supplementary Fig. 3c). Therefore, excessive PGE_2_ signaling via EP2 compromises survival to IAV in an age-dependent manner.

We next examined if EP2 blockade could alter the clinical response to IAV in aged mice when the blockade is administered during active infection to model a therapeutic solution rather than a prophylactic one. To this end, we began administration of 7 daily i.p. injections of the EP2 antagonist, or vehicle control, starting on the day of infection in aged C57BL/6 mice (Supplementary Fig. 5e). We found no differences in survival between the treated and control aged mice in this scenario (Supplementary Fig. 5f, survival tracked up to 16 dpi).

### Prophylactic blockade of PGE_2_ signaling through the EP2 receptor reduces influenza viral load and disease severity in aged mice

**Fig. 6:**
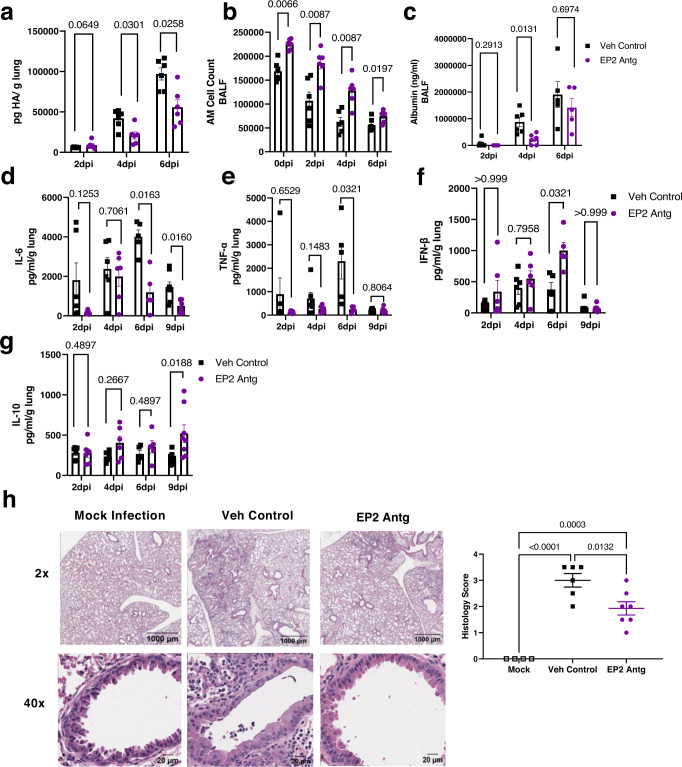
Prophylactic blockade of PGE_2_ signaling through the EP2 receptor reduces influenza viral load and disease severity in aged mice. Aged female C57BL/6 mice were given the EP2 antagonist or vehicle control by daily i.p. injection for seven days followed by intranasal (i.n.) infection of PR8 H1N1 as shown in Fig. 5a. Lung homogenate and BALF samples were collected over the course of the infection. **a** PR8 H1N1 IAV protein hemagglutinin (HA) measured in the lung homogenate on 2, 4, and 6 dpi by ELISA. *n* = 6/group. **b** AMs cell count in the BALF at 0, 2, 4, and 6 dpi. *n* = 6/group. **c** Albumin measured in the BALF on 2, 4, and 6 dpi. *n* = 7 for 2 dpi groups. *n* = 6/group for 4 dpi groups. *n* = 5/group for 6;dpi groups. TNF-α (**d**), IL-6 (**e**), IFN-β (**f**), and IL-10 (**g**) measured from the lung homogenate on 2, 4, 5, 9 dpi. For panels **d**–**g**, *n* = 6/group for 2 dpi groups and 4 dpi groups. *n* = 5/group for 6 dpi groups. *n* = 8/group for 9 dpi groups. For panels **a**–**g**, statistical significance analyzed by multiple Mann–Whitney tests with FDR correction, error bars represent SEM, and each point represents a biological replicate. **h** Representative images and scoring of H&E stained lungs at collected at 4 dpi along with quantification of histological score. *n* = 4 for Mock. *n* = 6 for Veh Control. *n* = 7 for EP2 Antg. Statistical significance analyzed by ANOVA with Tukey post hoc test, error bars represent SEM and each point represents a biological replicate. Source data are provided as a Source Data file.

Given that prophylactic EP2 receptor blockade increased survival of aged mice following IAV infection, we next examined if EP2 antagonism affected viral load. To monitor viral load, we treated aged C57BL/6 mice prophylactically with the EP2 antagonist or vehicle for 7 days, infected them with IAV, and measured the viral protein hemagglutinin (HA) in lung homogenates at various time points throughout the infection. EP2 antagonism significantly reduced viral HA protein levels by ~2-fold compared to the vehicle control at both 4 and 6 dpi (Fig. 6a). Similar to our observations that EP2 antagonism increased total AM cell numbers in non-infected aged mice, we observed an increase in AM cell numbers in BALF of EP2 antagonist-treated mice over the course of infection (Fig. 6b). Linear regression analysis showed that the viral load, as measured by HA protein levels, negatively correlated with AM numbers in the BALF during IAV infection (Supplementary Fig. 6a).

As PGE_2_ is a lipid known to regulate several immune cell types^48,49^, we characterized how prophylactic blockade of the EP2 receptor affects immune cell accumulation into the lung and BALF following IAV infection using flow cytometry (gating strategy shown in Supplementary Fig. 6b). Within the lung tissue, we observed no differences in the number of CD45^+^ hematopoietic (Supplementary Fig. 6c), CD4^+^ T cells (CD3^+^CD4^+^CD8^-^) (Supplementary Fig. 6D), CD8^+ T^ cells (CD3^+^CD4^-^CD8^+^) (Supplementary Fig. 6e), B cells (CD45^+^ B220^+^) (Supplementary Fig. 6f) and AEC II cells (CD45^-^EpCAM^+^) (Supplementary Fig. 6g). We also found no differences in neutrophil numbers in the BALF between EP2 antagonist vs. vehicle control treated groups (Supplementary Fig. 6h).

EP2 blockade reduced lung damage, inferred from a reduced level of albumin in the BALF, a marker of lung damage, at 4 dpi as compared to control (Fig. 6c). Aged mice treated with the EP2 antagonist exhibited ~2–3-fold reductions in the levels of the proinflammatory cytokines IL-6 (6 and 9 dpi) and TNF-α (6 dpi) (Fig. 6d, e). We also observed higher levels of the anti-viral cytokine interferon (IFN)-β in the EP2 antagonist-treated mice at 6 dpi (Fig. 6f). Mice begin to recover from infection and resolve the inflammation in the lungs at 9 dpi, and we found that the EP2 antagonist-treated mice exhibited a ~1.75-fold increase in the immunosuppressive cytokine IL-10 at 9 dpi (Fig. 6g), indicating that the EP2 antagonist-treated mice are better able to resolve the inflammation within the lungs than vehicle-treated counterparts. Mice treated with the EP2 antagonist also showed reduced histological evidence of inflammation at 4 dpi (Fig. 6h). Overall, these data indicated that the prophylactic EP2 antagonist treatment in aged mice leads to reduced viral load, inflammatory cytokines, and subsequent lung damage following infection.

## Discussion

The dysregulation of the immune system with age, called immunosenescence, contributes to increased morbidity and mortality to respiratory pathogens, including IAV^50^. While the effects of immunosenescence on adaptive immunity are well-studied, the effects of immunosenescence on resident innate immune cells are less well understood^50,51^. Here, we show that the overproduction of PGE_2_ with aging compromises host defense to IAV infection by impairing AMs, lung resident macrophages that are critical to both lung homeostasis and host defense^52^. Importantly, we show that blocking PGE_2_ signaling in aged, but not young, mice increase AM numbers prior to infection, and subsequently enhances the survival to lethal IAV infection. Our study demonstrates that blocking PGE_2_ signaling via inhibiting the EP2 receptor is sufficient to increase AM numbers via increased proliferation in non-infected aged mice and protects aged mice from lethal IAV infection by augmenting innate immune defense and inflammation resolution. This could explain several observations of our study, including that the EP2 receptor blockade in aged mice reduced viral burden, reduced inflammatory cytokines, increased anti-viral IFN-β levels, reduced markers of lung tissue damage, and importantly increased survival to lethal IAV infection with aging. Overall, our data suggest that the prophylactic EP2 receptor blockade improves host defense against IAV with aging.

Thus, age-elevated PGE_2_ is detrimental to host immunity against IAV infection. While our study focuses on the effects of PGE_2_ in the lung on AM-mediated immunity, we cannot exclude the effects of other age-associated factors within the lung that may also impair host defense against IAV, which require future investigations.

**Fig. 7:**
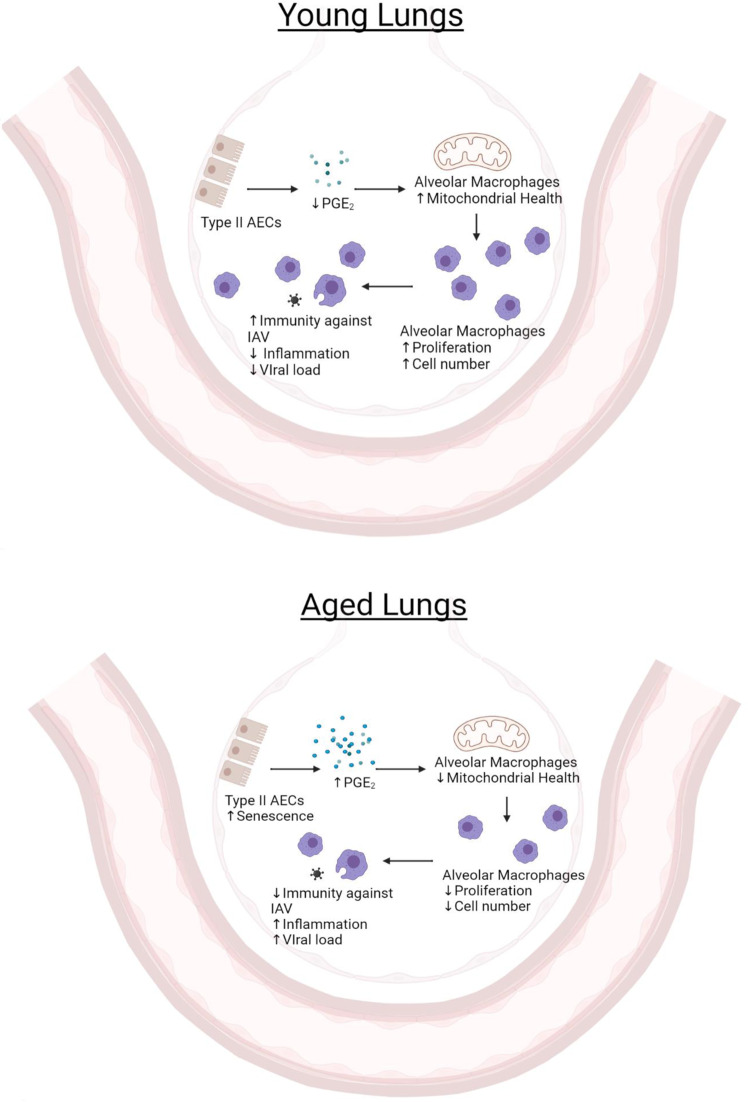
Aging leads to pathological communication between AECs and AMs via PGE_2_ secretion. Top: with youth there are low secreted concentrations of PGE_2_ by type II AECs which remain below the level that would negatively impact AM mitochondrial function. Hence, there are sufficient AM numbers to maintain homeostasis and provide host defense to influenza infection. Bottom: with aging, senescent type II AECs secrete increased levels of PGE_2_ that subsequently limit AM proliferation and mitochondrial function, thereby reducing AM numbers. Consequently, host defense to influenza viral infection is compromised. Schematics created in BioRender.

We identified senescent type II AECs as a major cellular source of PGE_2_ in the aged lung. Our study suggests that with aging, senescent type II AECs communicate aberrantly with AMs via excessive PGE_2_ secretion, which impairs AM mitochondrial function and proliferation, graphically illustrated in Fig. 7. While the canonically studied SASP factors are proteins such as cytokines, chemokines and growth factors, there is growing evidence that non-protein factors, such as lipids, are included in SASP^21,22,53^. Our study, along with others, suggest that PGE_2_ is a lipid SASP factor. We show that the removal of senescent cells, via senolytic treatment, in type II AECs from aged, but not young, mice reduced PGE_2_ secretion. The failure of senolytics to reduce PGE_2_ secretion of type II AECs from young mice may be explained by the lack of senescence, as measured by p21 expression and low secretion of IL-6 and TNF-α, in these cells.

Senescence modulates the secretome of cells in a context-dependent and cell type-dependent manner. We identified PGE_2_, IL-6, and TNF-α as part of the secretome of senescence type II AECs. However, there are numerous additional factors such as inflammatory cytokines, chemokines, immune-modulating factors, and growth factors that may also contribute to the secretome of senescent type II AECs^54,55^. Future investigation is needed to characterize the SASP secretome of type II AECs and how these factors modulate AMs and host defense against pulmonary infections with aging.

Interestingly, PGE_2_ induces and maintains senescence in fibroblasts^56^ and CD8^+^ T cells^57^. In addition, PGE_2_ can signal in an intracrine manner by acting on intracellular EP receptors^56^. Whether PGE_2_ participates in a positive-feedback loop with the senescence phenotype in type II AECs, or other cells in the lung, requires future investigation.

In the current study, we show that PGE_2_ increases in the BALF with aging in both mice and humans. Importantly, there is variability in the expression of PGE_2_ in humans with age. Little is known about how PGE_2_ levels is regulated in the BALF in humans, but studies have shown that cigarette smoke and nicotine exposure can modulate PGE_2_ levels in the lung^11,58^. The majority of PGE_2_ studies in humans have taken measurements from the plasma or serum. For example, plasma PGE_2_ levels correlate with C-reactive protein levels, type II diabetes, and plasma glucose levels in humans^59^. Whether or not factors that impact PGE_2_ levels in the plasma also impact PGE_2_ levels in the lung are not known. Future translational studies are needed to understand how PGE_2_ expression is modulated in the BALF in humans with aging, and also to identify the factors that contribute to the variability of PGE_2_ expression. Also given the complexities of the human immune system with aging, future studies will be required to determine the clinical impact of increased PGE_2_ levels in the lungs with aging.

Our results suggest that PGE_2_ restriction of AM proliferation likely impairs self-renewal of AMs within the aged lung. Our study, however, does not exclude the possibility of increased recruitment of monocytes into the airways and their differentiation into AMs when blocking EP2 signaling in aged mice, thus contributing to the increased number of AMs. However, since monocytes are typically recruited to the lung under inflammatory conditions rather than at steady state, we do not suspect that monocyte recruitment is a major contributor to the increased AMs identified prior to infection in our model^60,61^. In addition, blocking the EP2 receptor may reduce monocyte recruitment into the airways as studies using EP2 deficient mice show that PGE_2_-EP2 signaling is necessary for monocyte recruitment following myocardial injury^62^. In addition, the EP2 antagonist has a pharmacological half-life of less than 10 h in rodents^36^, therefore the antagonist is cleared systemically prior to the recruitment of monocytes into the lung, which occurs around 1 day post IAV infection^63^. Also, our prior work has shown that PGE_2_ impairs AM proliferation in vitro^11^. Thus, we believe that the increased number of AMs in aged mice given the EP2 antagonist is due to increased proliferation of AMs rather than recruitment of monocytes; however, further studies are necessary to understand how PGE_2_ regulates monocyte recruitment and differentiation into AMs.

We found that whereas EP2 receptor blockade increased AM numbers in non-infected aged mice, it failed to do so in non-infected young mice. Young mice, who have lower PGE_2_ levels relative to aged mice, may already have optimized PGE_2_ levels so that EP2 blockade is ineffectual at increasing AM numbers. We have previously shown that AMs are both critical to survival and limit lung injury following IAV infection in mice with aging^8^. In addition, we have shown that with aging AMs are further depleted post IAV infection^8^. Hence, it is likely that 7 days of prophylactic treatment of EP2 blockade sufficiently boosted AM numbers to improve outcomes after IAV infection in aged mice, whereas starting EP2 blockade on the day of infection likely did not provide sufficient time to increase AM numbers during IAV infection.

Our study also indicates that PGE_2_ perturbs the metabolic health of AMs via mitochondrial dysfunction, a hallmark of aging^16^. To our knowledge, our results show for the first time that PGE_2_ impairs mitophagy through the EP2 receptor. It remains possible that PGE_2_ also impacts mitochondrial health in AMs independently of its effects on mitophagy. Whether PGE_2_ restricts mitochondrial health, via mitophagy or other mechanisms, in other immune cells, and the possible physiological consequences thereof, will require future investigation.

It is unlikely that the role of PGE_2_ in lung aging uncovered here is specific to IAV infection and we expect that our findings will readily extend to other respiratory infections such as SARS-CoV-2, as aging is a prominent risk factor for severe COVID-19. Interestingly, COVID-19 severity is positively correlated with PGE_2_ serum levels^64^. In addition, infecting Calu-3 cells, a human lung epithelial cell line, with SARS-CoV-2 induced PGE_2_ production^64^; similar to our finding that IAV infection increases PGE_2_ levels in the lungs in vivo, and promotes PGE_2_ production by primary type II AECs ex vivo. Besides respiratory viruses, AMs also play critical roles in the immune defense against respiratory bacteria such as *Streptococcus pneumoniae* and *Staphylococcus aureus*^65–67^. The limiting of AM cell numbers by increased PGE_2_ in the lungs of older adults may play a role in the high incidence of pneumonia and pneumonia-related morbidity in older adults^68–70^. In addition to proliferation, PGE_2_ has been shown to limit AM phagocytosis, killing of bacteria and the expression of the toll-like receptor 4^33,34^. These may represent additional mechanisms by which age-enhanced PGE_2_ levels in the lungs limit AM-mediated immunity to IAV or other respiratory pathogens, which will be revealed through future study.

In conclusion, our study has revealed that age-associated overproduction of PGE_2_ in the lung, largely by senescent type II alveolar epithelial cells, impairs AM mitochondrial function, cellular proliferation, and reduces total AM cell numbers. This limits the ability of AMs to defend against IAV infection. Our study introduces new potential therapeutic targets—namely senescent type II AECs, their production of PGE_2_ and the EP2-mediated signaling in AMs—to reduce the burden of IAV, and possibly other respiratory viruses such as coronaviruses, in older adults.

## Methods

### Study approval

All animal experiments were approved prior to the initiation of the study and were carried out in accordance with the University of Michigan Institutional Animal Care and Use Committee. The use of de-identified lungs was provided by BK under protocol #23201 at Temple University USA. These samples were provided by the Gift of Life Program of Philadelphia, USA. Please note that informed consent is obtained from families of donors who have agreed to gift the family member’s organs to the Gift of Life Program of Philadelphia, USA. Also note that the IRB at Temple University has determined that as the human samples from the Gift of Life Program of Philadelphia are de-identified, the research from these samples is considered non-human subject research.

### Mice

Young (2–4 months) and aged (18–22 months) female C57BL/6N mice were obtained from Charles Rivers and the National Institute of Aging rodent facility at Charles Rivers. Male UM-HET3 mice were kindly gifted by Dr Richard Miller at the University of Michigan. The male UM-HET3 mice were aged at the Glenn Center on Aging at the University of Michigan. MitoQC mice were donated from the lab of Dr Ian Ganley at the University of Dundee and originally generated by Taconic Artemis^44^. The MitoQC mice were then bred and housed within the animal facility at the North Campus Research Complex at the University of Michigan. Male and female EP2 KO (*Ptger2* KO, Jackson Laboratory, strain #004376), on the C57BL/6 background, were obtained from the lab of Dr Marc Peters-Golden at the University of Michigan. All mice were maintained on a 12-h light-dark cycle with free access to food and water within a specific-pathogen-free facility. Mice were monitored for at least 1 week after arrival to our facilities for signs of stress and/or disease. Animals that displayed evidence of infection or illness prior to influenza infection were excluded from the study.

### PGE_2_ receptor antagonists in vivo

The EP2 antagonist PF-04418948 (catalog #15016) and the EP4 antagonist ONO-AE3-208 (catalog #14522) were purchased from Cayman Chemical. Mice were given daily doses of 10 mg/kg EP2 and/or 10 mg/kg EP4 antagonist(s) by intraperitoneal (i.p.) injections. For experiments treating mice with the EP2 and EP4 antagonists starting on the day of influenza infection, mice were given daily injections on day 0 to day +7 post infection. For experiments treating mice with the EP2 and EP4 antagonists both prior to and during influenza infection, mice were given 11 total daily injections starting on day −7 pre infection and up to day +4 post infection. For experiments treating mice with the EP2 antagonist prior to influenza infection, mice were given daily injections on day −7 to day 0 pre infection.

### Virus

Stocks of the VR-95 Influenza A/PR8/34 H1N1 were purchased from ATCC.

### In vivo influenza infection

Mice were anesthetized with isoflurane and instilled with 400 pfu of influenza A virus in 40 µL PBS or 40 µL PBS vehicle control. Mice were euthanized after loss of 30% of their pre-infection body weight.

### In vitro influenza infection

Cells were infected with an MOI = 0.1 of influenza virus diluted in cell culture medium for 48 h.

### Sample collection and preparation

Bronchoalveolar lavage fluid (BALF): the BALF was collected by lavaging the lungs twice with 1 mL of cold sterile PBS. Cells in the BALF were resuspended into FACS buffer (PBS + 2 mM EDTA + 4% FBS) for flow cytometry staining or RPMI-1640 medium containing 10% FBS and 100 U/mL penicillin/streptomycin for ex vivo culture.

Lung homogenate: lungs were homogenized using a TissueLyser II (Qiagen). Following, the samples were centrifuged and the supernatant was collected.

Lung single-cell suspension: lungs were digested with 1 mg/mL Collagenase D (Roche, COLLD-RO) and 10 U/mL DNase (Roche, 04536282001) for 45 min at room temperature with agitation. After digestion, the lungs were minced and passaged through a 100 µm cell strainer. To remove red blood cells, cells were incubated with red blood cell lysis buffer (BioLegend, 420301) for 3 min.

### Flow cytometry

Cells were obtained from the BALF, single-cell suspension of lungs or cell culture. Flow cytometry was performed using the ZE5 Cell Analyzer (BioRad) of the Flow Cytometry Core at the University of Michigan. Analysis of flow cytometry data was performed using FlowJo (version 10.8.0).

Mitochondrial analysis: live cells were stained with MitoTracker DeepRed RM (ThermoFisher, M22426) to measure mitochondrial mass, tetramethylrhodamine methyl ester (TMRM) (ThermoFisher, T668) to measure mitochondrial membrane potential, and MitoSOX (ThermoFisher, M36008) to measure mitochondrial ROS, according to the manufacturer’s instructions.

Staining: cells were stained with a live/dead viability dye according to manufacturer’s instructions (ThermoFisher, L34966). Next, Fc blocking was performed by incubation with anti-CD16/32 antibody for 20 min (BioLegend, 101320). Following, cells were stained with the desired surface markers and fixed in 4% paraformaldehyde for 25 min. Cells were washed twice and resuspended in FACS buffer until analysis.

Antibodies: APC: BrdU (BD, cat# BD552598, dilution 1:50), CD45 (BioLegend, cat# 103112, clone 30-F11, dilution 1:100), Ki67 (BioLegend, cat# 6524065, clone 16A8, dilution 1:100); APC-eFluor 780: CD8 (eBioScience, cat# 14-0081-82, clone 53-6.7, dilution 1:100); Brilliant Violet 421: CD11b (BioLegend, cat# 101236, clone M1/70, dilution 1:100), CD3 (BioLegend, cat# 100228, clone 17A2, dilution 1:100), EpCam (BioLegend, cat# 118225, clone G8.8, dilution 1:100); Brilliant Violet 605: B220 (BioLegend, cat# 103244, clone RA3-6B2, dilution 1:100), Ly6G (BioLegend, cat# 127639, clone 1A8, dilution 1:100), CD11c (BioLegend, cat# 117334, clone N418, dilution 1:100); Brilliant Violet 750: SiglecF (BD, cat# BDB747316, clone E50-2440, dilution 1:100); Brilliant Violet 785: MHC II (BioLegend, cat# 107645, clone M5/114.15.2, dilution 1:100), FITC: Annexin V (R&D Systems, cat# 4830-01-K, dilution 1:100); PE: F4/80 (BioLegend, cat# 123110, clone BM8, dilution 1:100), SiglecF (BD, cat# BDB552126, clone E50-2440, dilution 1:100); PE-Cy7: CD4 (Invitrogen, cat# 25-0041-82, clone GK1.5, dilution 1:100); PE-eFluor 610: CD11c (Invitrogen, cat# 61-0114-82, clone N418, dilution 1:100).

### MH-S cell culture

MH-S cells, an immortalized mouse alveolar macrophage cell line^46^, were obtained from ATCC (ATCC, CLR-2019) and grown in RPMI-1640 medium containing 10% FBS, 0.05 mM 2-mercaptoethanol, and 100U/mL penicillin/streptomycin. For PGE_2_ stimulation assays, cells were cultured with 1 µM PGE_2_, 10 µM PGE2, or vehicle control, for 24 h.

### Isolation and culture of type II AECs

AECs were isolated by magnetic associated cell sorting (MACS) by negative selection of CD45^+^ (Miltenyi, cat#130-052-301, dilution 10 µL per 10 million cells) and CD31^+^ (Miltenyi, cat#130-097-41, dilution 10 µL per 10 million cells) cells and followed by positive selection of CD326^+^ cells (Miltenyi, cat# 130-118-075, clone caa7-9G8, dilution 5 µL per 10 million cells). This method typically yields approximately 85% purity of prosurfactant-C^+^ cells as measured by flow cytometry. Isolated type II AECs were cultured with DMEM/F12 medium containing 10% FBS, 1.25 g BSA, 100 U/mL penicillin/streptomycin, and 1x Insulin-Transferrin-Selenium (Gibco, 41400045). Type II AECs were seeded in tissue culture plates coated with gelatin-based coating solution (Cell Biologics, 6950).

To deplete senescent cells, type II AECs were cultured with 20 nM dasatinib (Sigma, SML2589) and 5 µM quercetin (Sigma, Q4951) or 200 nM dasatinib and 50 µM quercetin for 48 h.

### Seahorse assay

Seahorse experiments were conducted using a Seahorse XFe96 Analyzer (Agilent). Four basal readings were taken prior to the addition of the electron transport chain inhibitors in Agilent’s mitostress test: 1.5 µM oligomycin, 1 µM FCCP, and 0.5 µM rotenone and antimycin A (Agilent, 103708). Oxygen consumption rate (OCR) and extracellular acidification rate (ECAR) data were normalized to cell count. Seahorse experiments were repeated three times.

### BrdU in vivo

BrdU (Biogems, 5911439) was given to mice in their drinking water ad libitum for a total of 7 days. BrdU was dissolved in the drinking water at a concentration of 0.8 mg/mL and the BrdU water was refreshed every 48 h.

### Enzyme-linked immunosorbent assay (ELISA)

TNF-α (88-7324-22), IL-6 (88-7064-88), IFN-γ (88-7314-22) and IL-10 (88-7105-22) ELISA kits were purchased from Invitrogen; albumin ELISA kits were purchased from Abcam (ab108792); cyclic AMP (581001) and PGE_2_ (514010) ELISA kits were purchased from Cayman Chemical; IFN-β ELISA (42410) kits were purchased from pbl Assay Science; Influenza A/PR/8/1934 hemagglutinin (SEK11684) ELISA kits were purchased from SinoBiological, and PGE_2_ (MBS266212) ELISA kits were purchased from MyBioSource. All ELISAs were performed according to manufacturer’s directions. Each sample was analyzed with at least two dilutions and with two technical replicates per dilution.

### Immunoblotting

Type II AECs were isolated from young and aged mice as described above. Cells were lysed using cold RIPA buffer (Sigma, 89900) containing 1% protease inhibitor cocktail (Sigma, P8340) and phosphatase inhibitor cocktail (Sigma, P5726). Samples were denatured and reduced using NuPAGE LDS Sample Buffer (ThermoFisher, NP0007) and reducing agent (ThermoFisher, NP0009) according to manufacturer’s instructions. Lysates were electrophoresed on 4–12% gradient Bis-Tris gel (ThermoFisher, NP0322BOX) and transferred to 0.2 micron PVDF membranes (ThermoFisher, IB401001). The membranes were cut based on the expected molecular weight of proteins of interest prior to incubation with antibodies. The cut membranes were blotted for p21 (Santa Cruz, cat# sc-6246, clone F-5, dilution 1:200) and the loading control GAPDH (Cell Signaling Technology, cat# 2118S, clone 14C10, dilution 1:1000) or β-tubulin (Cell Signaling Technology, cat# 2146, dilution 1:1000). Blots were imaged using a BioRad ChemiDoc XRS+.

### Histology

Lung samples were fixed with 10% phosphate buffered formalin. Samples were submitted to the In Vivo Animal Core at the University of Michigan for paraffin embedding, sectioning, and H&E staining. The slides were scored by blinded investigators for lung damage, inflammation and cellular infiltration on a scale of 0–5 (0, being no damage or inflammation; 1, minimal evidence of inflammation; 2, moderate evidence of inflammation but limited tissue damage and few immune cell infiltration; 3, moderate of inflammation, tissue damage, and immune cell infiltration; 4, strong evidence of inflammation, tissue damage and immune cell infiltration; 5, severe damage and inflammation with strong evidence of immune cell infiltration).

### Transcriptomics analysis

A publicly available RNA-seq (GEO GSE134397) dataset of young and aged AM transcripts was downloaded from the Gene Expression Omnibus (GEO). Raw counts were processed in R (version 4.1.0) using edgeR (version 3.34.1) to generate TMM normalized counts^71^. False detection rate (FDR) *q* values (adjusted *p* values) were used to correct for multiple comparisons. A value of FDR = 0.05 was used as a threshold for statistical significance. Publicly available scRNA-seq dataset of type I and type II AEC transcripts (GSE113049, https://www.ncbi.nlm.nih.gov/geo/query/acc.cgi?acc=GSE113049) was downloaded from GEO and analyzed in Seurat (version 2.3.0)^25^. Unique molecular identifier (UMI) gene count matrixes were generated per sample and UMI counts were normalized to library size, scaled by 10,000 and log-transformed. Principal component analysis was performed on the *Z* scores of the normalized expression values. Cell type clustering was performed using the top 15 principal components and a resolution of 0.6.Tabula Muris scRNA-seq gene counts and analysis codes were accessed online on Figshare and GEO (GSE109774, https://www.ncbi.nlm.nih.gov/geo/query/acc.cgi?acc=GSE109774)^23^.

### Transmission electron microscopy (TEM)

AMs were cultured on 35 mm glass bottom dishes (MatTek, P35G-1.5-10-C) and then fixed with 2.5% glutaraldehyde in 0.1 M sodium cacodylate. Samples were then embedded in resin for sectioning and stained with heavy metals at the University of Michigan Microscopy Core. 2 micron sections were imaged on a JEM-1400Plus (JOEL) at ×5000 magnification. Images of 15 cells per condition and 10 mitochondrial per cell were captured.

### AM adoptive transfer

Mice were given 50 µL of clodronate-loaded liposomes (Encapsula Nano Sciences, CLD-8901) i.n. to deplete resident AMs prior to adoptive transfer. 36 h following AM depletion, 2 × 10^5^ sex-matched AMs in 40 µL of PBS were given intranasally.

### Human samples

Lungs from donors that were not used for transplantation were lavaged with HEPES-buffered saline and 2 mM EDTA. The fluid was centrifuged, and the supernatant was used for analysis.

### Statistics

All murine experiments were performed with at least four biological replicates. Experiments performed with immortalized cell lines were performed with at least three technical replicates. All results are presented as mean ± standard error of the mean (SEM). Data were analyzed using the nonparametric two-tailed Mann–Whitney with FDR correction for multiple tests when necessary, unless otherwise stated. Multiple comparison testing was done by ANOVA with Tukey post hoc. Survival differences were analyzed using a Gehan–Breslow–Wilcoxon test. Specific statistical tests are denoted in the figure legends. Two-sided *p* values <0.05 were considered significant. Prism 9 (GraphPad) was used for statistical testing and the generation of graphs.

### Reporting summary

Further information on research design is available in the Nature Portfolio Reporting Summary linked to this article.

## Supplementary information

Supplementary Information

Reporting Summary

## Data Availability

All source data generated during this study are provided with this article. Source Data are provided with this paper.

## Source data

Source Data
