# Supplementary material for: Age-induced prostaglandin E2 impairs mitochondrial fitness and increases mortality to influenza infection
Source: Nat Commun. 2022 Nov 9;13:6759. doi: 10.1038/s41467-022-34593-y (PMC9643978; doi:10.1038/s41467-022-34593-y)
Supplement: Supplementary file 1 — Supplementary Information [file 41467_2022_34593_MOESM1_ESM.pdf]

Supplemental Information

Supplemental Figures

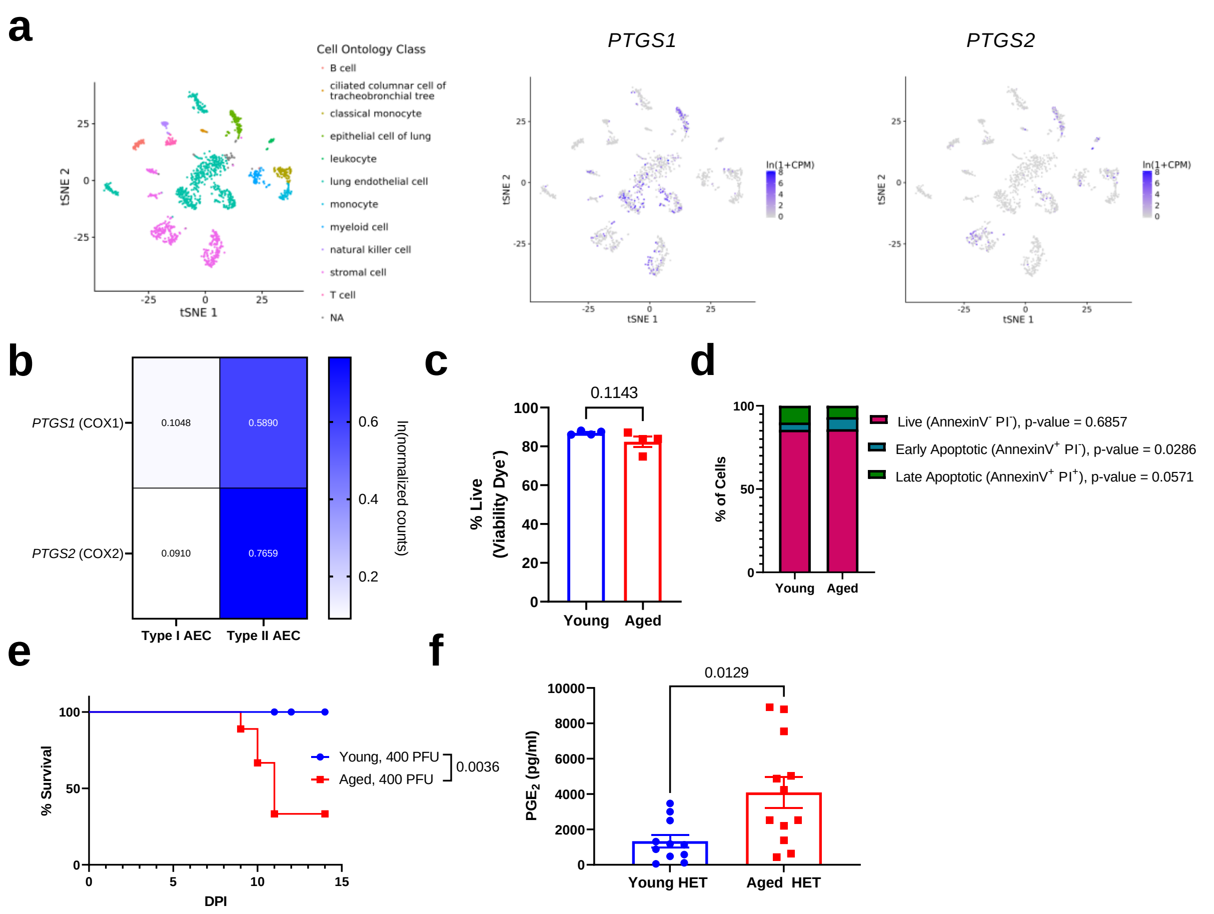

Supplemental Figure 1: (**a**) tSNE maps showing expression of *PTGS1* and *PTGS2* (**b**) Heatmap of the ln(normalized counts) of *PTGS1* (gene product = COX1) and *PTGS2* (gene product = COX2) of type I and type II AECs (GEO GSE113049). (**c**-**d**) Type II AECs were isolated from young and aged mice and cultured for 3 days. Cells were stained with (**c**) an amine-reactive viability dye to quantify live cells and (**d**) with AnnexinV and propidium iodide to quantify live, early apoptotic, and late apoptotic cells by flow cytometry. For Supplemental Figures 1c-d: statistical significance analyzed by Mann-Whitney tests, error bars represent SEM, each point represents a biological replicate, and n=4/group (**e**) Percent survival of young (2-4 months) and aged (18-22 months) female C57BL/6 mice infected with 400 pfu of IAV i.n. Survival differences were statistically determined by a two-tailed Gehan-Breslow-Wilcoxon test and n=9/group. (**f**) PGE_2 levels in the BALF of young (6 months) and aged (22 months) non-infected male UM-HET3 mice. Data analyzed by Mann-Whitney, error bars represent SEM, each point represents a biological replicate, n= 11 for Young HET, n=12 for Aged HET. Source data are provided as a source data file.

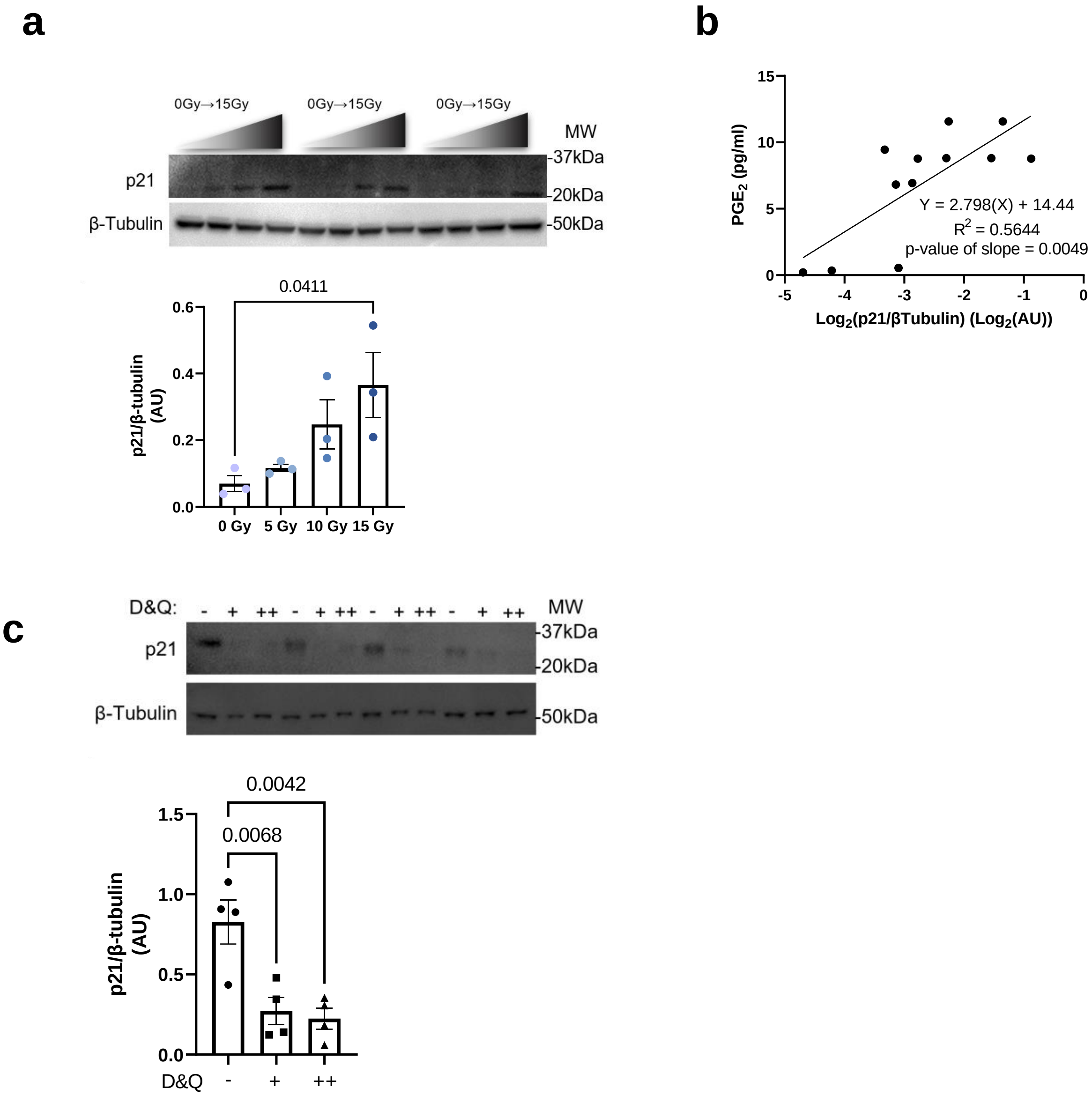

Supplemental Figure 2: (**a-b**) Type II AECs from young mice were isolated and irradiated with 0, 5, 10, and 15 Gy to induced senescence. (**a**) Expression of p21 was measured by western blot. Statistical significance analyzed by ANOVA with Tukey post-hoc test, error bars represent SEM, each point represents a biological replicate, and n=3/group. (**b**) PGE_2 was measured from the culture medium by ELISA. PGE_2 levels was plotted against log-transformed, β-tubulin normalized, p21 expression and a simple linear regression analysis was performed. The p-value of the slope was calculated based on the null hypothesis of slope = 0 and the alternative hypothesis of slope ≠ 0. Each point represents a biological replicate and n=12. (**c**) Type II AECs from aged mice were isolated and cultured with 20nM/5µM D&Q (+), 200nM/50µM D&Q (++) or vehicle control (-) for 48 hours. Expression of p21 was measured by western blot. Statistical

significance analyzed by ANOVA with Tukey post-hoc test, error bars represent SEM, each point represents a biological replicate, and n=4/group. Source data are provided as a source data file.

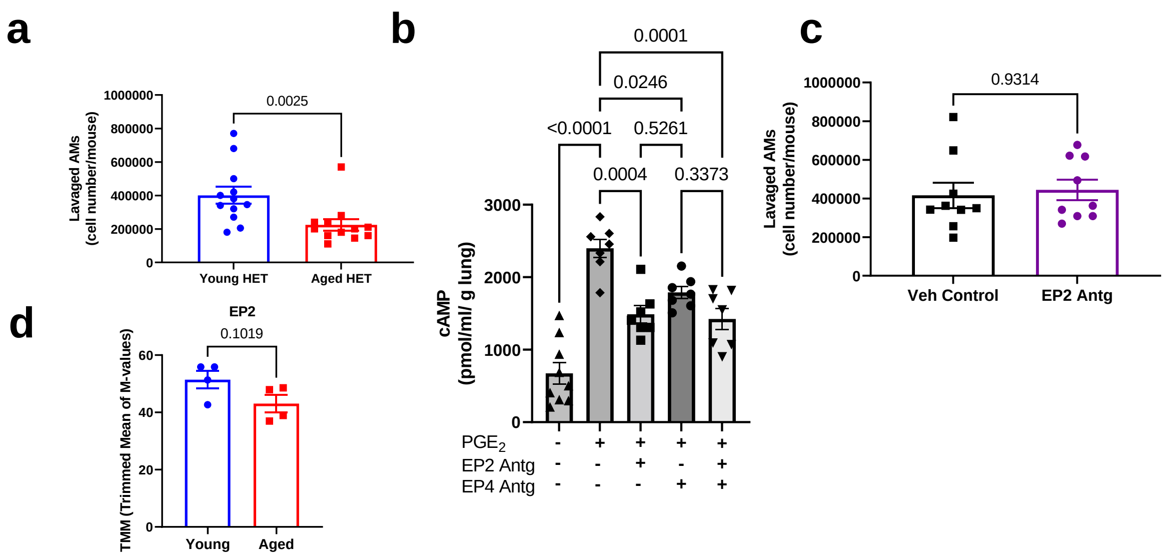

Supplemental Figure 3: (**a**) AMs in the BALF of young (6 months) and aged (22 months) male UM-HET3 mice were counted by flow cytometry. Statistical significance analyzed by Mann-Whitney, error bars represent SEM, each point represents a biological replicate and n=12/group. (**b**) Young (i.e., 2-4 months) C57BL/6 mice were given an i.p. injection of the EP2 antagonist and/or the EP4 antagonist or vehicle control followed by i.n. dosing of 15µg of PGE_2. Whole lungs were harvested 1 hour later. cAMP was measured in lung homogenate via ELISA. Statistical significance analyzed by ANOVA with Tukey post-hoc test, error bars represent SEM, each point represents a biological replicate, n=9 for negative control, n=7 for PGE_2 only, n= 7 for EP2 Antg + PGE_2, n=7 for EP4 Antg + PGE_2, n=7 for EP2 Antg+EP4 Antg+PGE_2 (**c**) Young (i.e., 2-4 months) female C57BL/6 mice were given 7 daily i.p. injections of 10mg/kg EP2 antagonist. AMs (i.e., $CD45^+$ $CD11c^+$ $SiglecF^+$) were then collected from the BALF and enumerated by flow cytometry. n=9/group. (**d**) Trimmed mean of M-values (TMM) normalized counts of EP2 of AMs sorted from young and aged C57BL/6 mice (GEO GSE134397). n=4/group. For Supplemental Figures 3c-d: statistical significance analyzed by Mann-Whitney, error bars represent SEM, and each point represents a biological replicate. Source data are provided as a source data file.

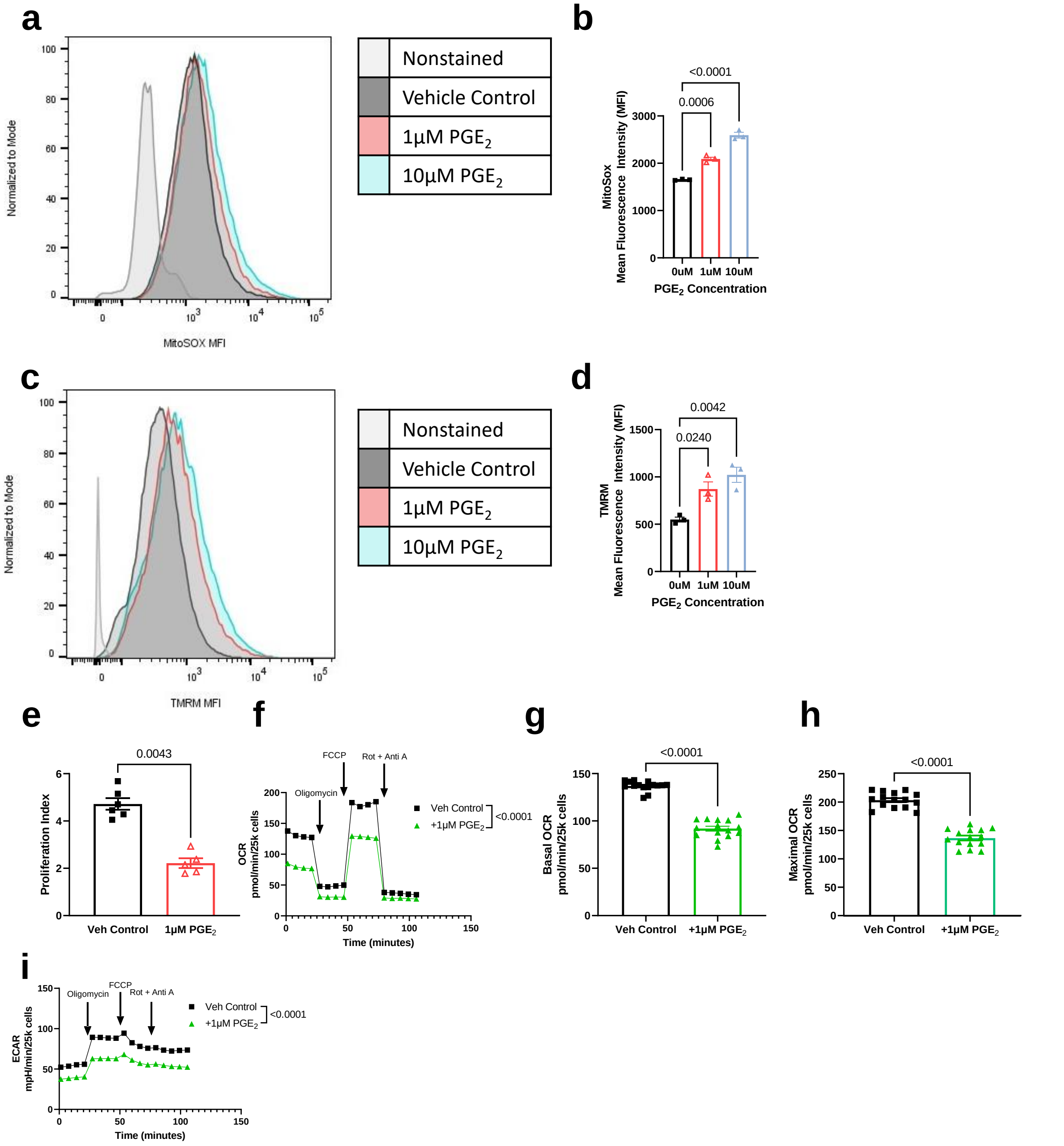

Supplemental Figure 4: MH-S cells were cultured with varying concentrations of PGE_2 for 24 hours. Cells were than stained with (**a-b**) MitoSox and (**c-d**) TMRM and analyzed by flow cytometry. Mean fluorescence intensity (MFI) was quantified. For Supplemental Figures 4b and 4d: statistical significance analyzed by ANOVA with Tukey post-hoc test, error bars represent SEM, each point represents a biological replicate, and n=3/group. (**e**) Proliferation index of MH-S cells were obtained by counting cells under a hemocytometer and calculating proliferation index as (total cells) / (number of cells seeded). Statistical significance analyzed by Mann-Whitney, error bars represent SEM, each point represents a replicate, n=6 for Vehicle Control and n=5 for 1µM PGE_2. (**f-i**)

MH-S cells were cultured for 24 hours with 1µM PGE_2 or vehicle control. OCR and ECAR were analyzed by a Seahorse xFe96 analyzer. (**f**) OCR measurements (**g**) Basal OCR measurements (**h**) Maximal OCR measurements (**i**) ECAR measurements. For Supplemental Figures 4f and 4i: statistical significance analyzed by ANOVA with Tukey post-hoc test and error bars represent SEM.
For Supplemental Figures 4g-h: statistical significance analyzed by Mann-Whitney, error bars represent SEM, each point represents one biological replicate, and n=15/group. Source data are provided as a source data file.

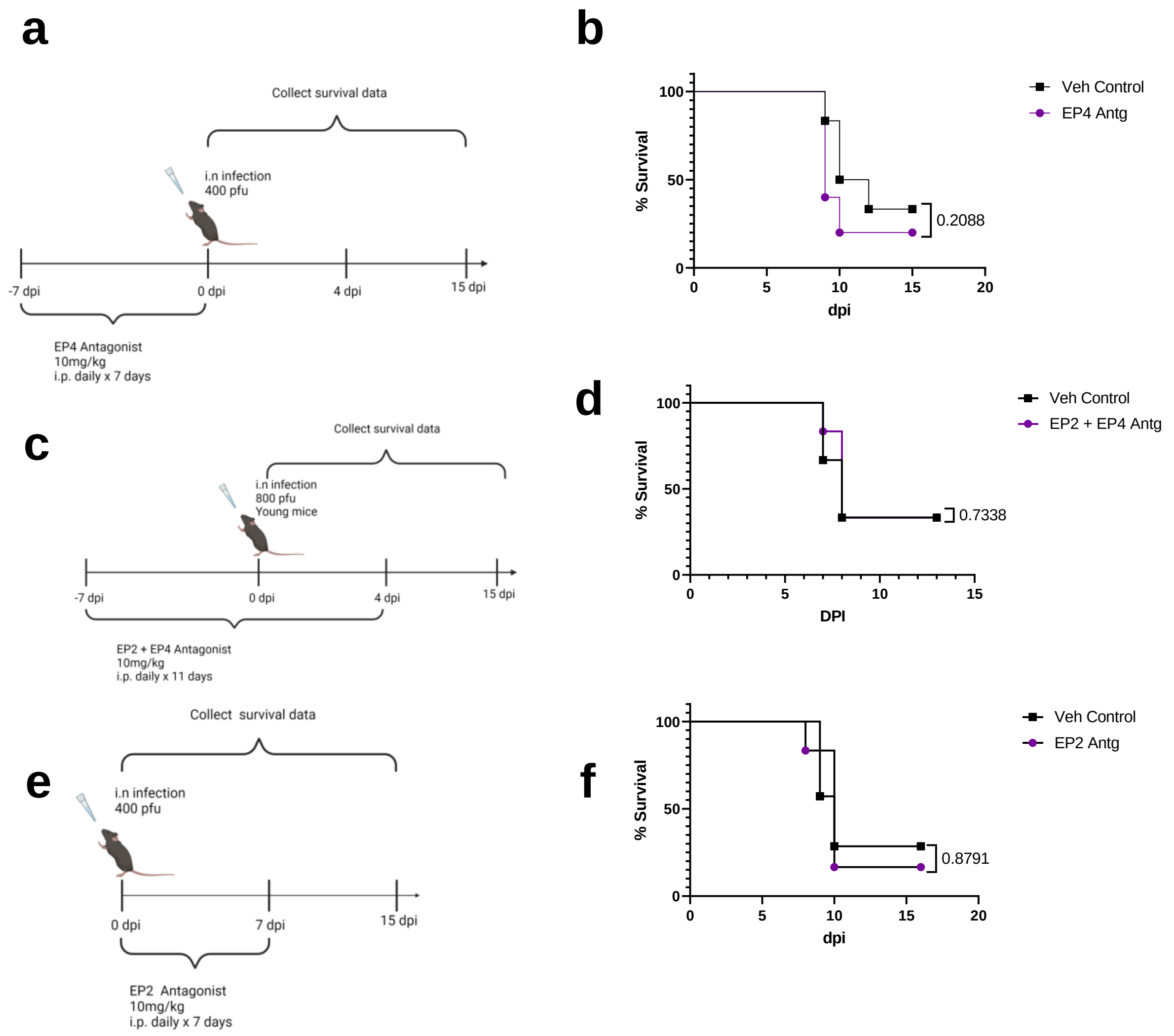

Supplemental Figure 5: (**a-b**) Aged C57BL/6 female mice were prophylactically treated with the EP4 antagonist or vehicle control by daily i.p. injections for 1 week pre-infection as showin in (**a**). Survival (**b**) was tracked daily. n=6 for Veh Control and n=5 for EP4 Antg. (**c-d**) Young female C57BL/6 mice were given the EP2 antagonist and EP4 antagonist or vehicle and infected with 800pfu of PR8 H1N1 as shown in (**c**). Survival of mice (**d**) were recorded daily. n= 6/group. (**e-f**) Aged C57BL/6 mice were treated with the EP2 antagonist or vehicle control by daily i.p. injections for 1 week starting on the day of infection as shown in (**e**). Survival of mice (**f**) were tracked daily. n=7 for Veh Control and n=6 for EP2 Antg. For Supplemental Figures 5b, 5d, 5f: survival differences were statistically determined a two-tailed Gehan-Breslow-Wilcoxon test**.** Schematics created in BioRender. Source data are provided as a source data file.

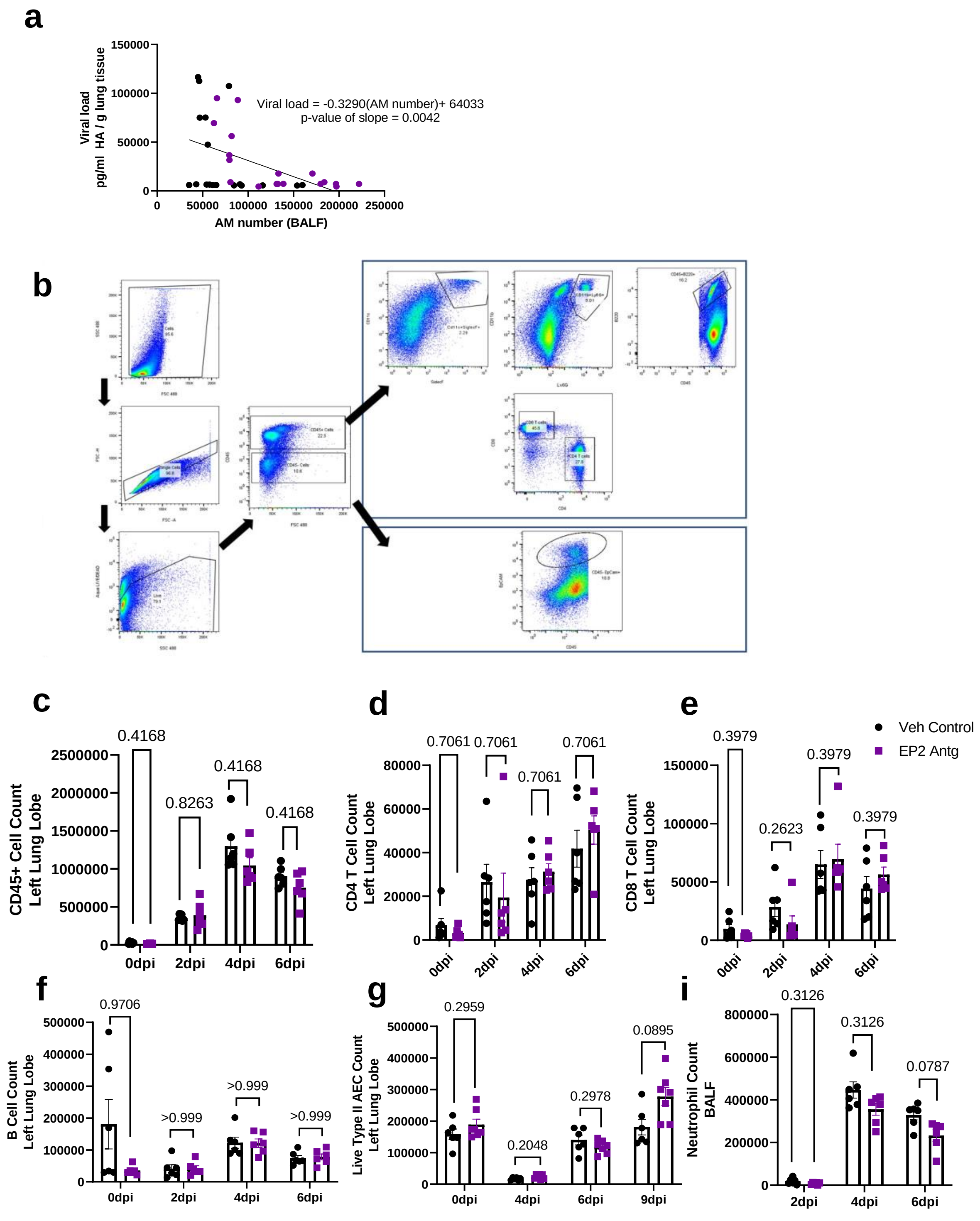

Supplemental Figure 6: (**a**) Aged C57BL/6 mice were prophylactically treated with the EP2 antagonist or vehicle control by daily i.p. injections for 1 week prior to infection with 400pfu of IAV. Linear regression analysis of AM number measured from the BALF and HA protein measured from the lung homogenate. Data points were copied from Figures

6A-B. Each point represents a biological replicate and n=36. (**b-h**) Flow cytometric analysis of immune cells of vehicle or EP2 antagonist treated aged C57BL/6 mice over the course of IAV infection. (**b**) Gating strategy (**c**) Total cell count of hematopoietic ($CD45^{+}$) cells. **(d**) Number of $CD4^{+}$ T cells ($CD3^{+}$ $CD4^{+}$ $CD8^{-}$) (**e**) Number of $CD8^{+}$ cells (i.e., $CD45^{+}$ $B220^{+}$) (**f**) Number of B cells ($CD45^{+}$ $B220^{-}$) (**g**) Total type II AEC cell count (i.e.,$CD45^{-}$ $EpCAM^{+}$) (**h**) Number of neutrophils ($CD45^{+}$ $Ly6G^{+}$). For Supplemental Figures 6C-H: Statistical significance analyzed by Mann-Whitney with FDR correction, error bars represent SEM, each point represents one biological replicate, and n=6/group. Source data are provided as a source data file.

Supplemental Tables

			Smoking Status			
**Variable**	**All Samples**		**NonSmokers**	**ExSmokers**	**Smokers**	**p-value**
**n (%)**	22		7 (31.8%)	8 (36.3%)	7 (31.8%)	
**Age (SD)**	43.7 (25.6)		36.9 (32.7)	56.6 (20.7)	35.7 (19.2%)	0.204
**Sex**						0.533
- Female, n (%)	12 (54.5%)		5 (71.4%)	4 (50%)	3 (42.8%)	
- Male, n (%)	10 (45.5%)		2 (28.6%)	4 (50%)	4 (57.2%)	
**Ethnicity**						0.605
- Caucasian, n (%)	16 (72.7%)		5 (71.4%)	7 (87.5%)	4 (57.1%)	
- African American, n (%)	3 (13.6%)		1 (14.3%)	1 (12.5%)	1 (14.3%)	
- Hispanic, n (%)	3 (13.6%)		1 (14.3%)	0 (0%)	2 (28.6%)	

Supplemental Table 1: Demographics of healthy human BALF samples. Age variable summarized with mean and standard deviation (SD). The categorical variables are summarized by count (n) and proportions. The summary information is given for all samples and by the smoking status. ANOVA was used to test for a difference in age across all three groups. Chi-Square test was used to test for a difference in the categorical variables (sex and ethnicity) across all three groups.
