# Supplementary material for: Age-induced prostaglandin E2 impairs mitochondrial fitness and increases mortality to influenza infection
Source: Nat Commun. 2022 Nov 9;13:6759. doi: 10.1038/s41467-022-34593-y (PMC9643978; doi:10.1038/s41467-022-34593-y)
Supplement: Supplementary file 2 — Reporting Summary [file 41467_2022_34593_MOESM2_ESM.pdf]

nature portfolio

Corresponding author(s): Daniel R. Goldstein

Last updated by author(s): 10/25/2022

Reporting Summary

Nature Portfolio wishes to improve the reproducibility of the work that we publish. This form provides structure for consistency and transparency in reporting. For further information on Nature Portfolio policies, see our Editorial Policies and the Editorial Policy Checklist.

Statistics

For all statistical analyses, confirm that the following items are present in the figure legend, table legend, main text, or Methods section.

n/a	Confirmed	
☐	☒	The exact sample size (*n*) for each experimental group/condition, given as a discrete number and unit of measurement
☐	☒	A statement on whether measurements were taken from distinct samples or whether the same sample was measured repeatedly
☐	☒	The statistical test(s) used AND whether they are one- or two-sided<br>*Only common tests should be described solely by name; describe more complex techniques in the Methods section.*
☐	☒	A description of all covariates tested
☐	☒	A description of any assumptions or corrections, such as tests of normality and adjustment for multiple comparisons
☐	☒	A full description of the statistical parameters including central tendency (e.g. means) or other basic estimates (e.g. regression coefficient) AND variation (e.g. standard deviation) or associated estimates of uncertainty (e.g. confidence intervals)
☐	☒	For null hypothesis testing, the test statistic (e.g. *F*, *t*, *r*) with confidence intervals, effect sizes, degrees of freedom and *P* value noted<br>*Give P values as exact values whenever suitable.*
☒	☐	For Bayesian analysis, information on the choice of priors and Markov chain Monte Carlo settings
☒	☐	For hierarchical and complex designs, identification of the appropriate level for tests and full reporting of outcomes
☒	☐	Estimates of effect sizes (e.g. Cohen's *d*, Pearson's *r*), indicating how they were calculated

Our web collection on statistics for biologists contains articles on many of the points above.

Software and code

Policy information about availability of computer code

Data collection	Seahorse experimental data was collected using a Seahorse XF Analyzer and the Seahorse Wave Desktop Software from Agilent.<br>Flow cytometry experiments were performed using the ZE5 Cell Analyzer and the Everest Software (ver 2.5.0.10) from BioRad.
Data analysis	Statistical analysis performed in Graphpad Prism (version 9.0). Flow cytometry data analyzed in FlowJo (version 10.8.0). For transcriptomics analysis, R (version 4.1.0), edgeR (version 3.34.1), and Seurat (version 2.3.0) were used.

For manuscripts utilizing custom algorithms or software that are central to the research but not yet described in published literature, software must be made available to editors and reviewers. We strongly encourage code deposition in a community repository (e.g. GitHub). See the Nature Portfolio guidelines for submitting code & software for further information.

Data

Policy information about availability of data

All manuscripts must include a data availability statement. This statement should provide the following information, where applicable:

- Accession codes, unique identifiers, or web links for publicly available datasets
- A description of any restrictions on data availability
- For clinical datasets or third party data, please ensure that the statement adheres to our policy

Source data for all figures are provided with the paper.

Field-specific reporting

Please select the one below that is the best fit for your research. If you are not sure, read the appropriate sections before making your selection.

☒ Life sciences ☐ Behavioural & social sciences ☐ Ecological, evolutionary & environmental sciences

For a reference copy of the document with all sections, see nature.com/documents/nr-reporting-summary-flat.pdf

Life sciences study design

All studies must disclose on these points even when the disclosure is negative.

Sample size	Sample size was determined based on prior studies involving aging and influenza from our lab (Wong et al, JI, 2017; Kulkarni et al, Mucosal Immunology, 2019; Smith et al, Aging Cell, 2019; Smith et al, JCI Insight, 2019)
Data exclusions	Animals that displayed evidence of infection or illness prior to influenza infection were excluded from the study
Replication	To ensure reproducibility, all experiments were performed with at least two technical replicates. For ELISAs, all samples were analzyed with at least two dilutions and with at least two technical replicates per dilution
Randomization	Allocation for experimental groups were done randomly.
Blinding	No blinding was done in this study. Influenza-infected samples and mice had to be properly labeled for laboratory safety

Reporting for specific materials, systems and methods

We require information from authors about some types of materials, experimental systems and methods used in many studies. Here, indicate whether each material, system or method listed is relevant to your study. If you are not sure if a list item applies to your research, read the appropriate section before selecting a response.

Materials & experimental systems

n/a	Involved in the study	
☐	☒	Antibodies
☐	☒	Eukaryotic cell lines
☒	☐	Palaeontology and archaeology
☐	☒	Animals and other organisms
☐	☒	Human research participants
☒	☐	Clinical data
☒	☐	Dual use research of concern

Methods

n/a	Involved in the study	
☒	☐	ChIP-seq
☐	☒	Flow cytometry
☒	☐	MRI-based neuroimaging

Antibodies

Antibodies used

1. anti-p21 (Santa Cruz, Cat# sc-6246, clone F-5, dilution 1:200, lot: L2921)
2. anti-GAPDH (Cell Signaling Technology, cat# 2118s, clone 14C10, dilution 1:1000, lot: 14)
3. anti-betaTubulin (Cell Signaling Technology, Cat# 2146S, dilution 1:1000, lot: 9)
4. anti-CD45 Microbeads (Miltenyi, Cat #130-052-301, dilution 10ul per 10 million cells, lot: 5200507048)
5. anti-CD31 Microbeads (Miltenyi, Cat # 130-097-418, dilution 10ul per 10 million cells, lot: 5200700545)
6. anti-CD326 (Miltenyi, cat# 130-118-075, clone caa7-9G8, dilution 5ul per 10 million cells, lot: 1321021057)
7. anti-BrdU - APC (BD, Cat# BD552598, dilution 1:50, lot: 0041156)
8. anti-CD45 - APC (Biolegend, cat# 103112, clone 30-F11, dilution 1:100, lot: B289585)
9. anti-Ki67 – APC (Biolegend, cat#652405, clone 16A8, dilution 1:100, lot: B278300)
10. anti-CD8 – APCeFluor780 (eBioScience, cat# 14-0081-82, dilution 1:100, clone 53-6.7, lot: 4322567)
11. anti-CD11b- BV 421 (Biolegend, cat# 101235, clone M1/70, dilution 1:100, lot: B303400)
12. anti-CD3- BV421 (Biolegend, cat# 100227, clone 17A2, dilution 1:100, lot: B343124)
13. anti-EpCam- BV421 (Biolegend, cat# 118225, clone G8.8, dilution 1:100, lot: B265073)
14. anti-B220 – BV605 (Biolegend, cat# 103243, clone RA3-6B2, dilution 1:100, lot: B310744)
15. anti-Ly6G- BV605 (Biolegend, cat# 127639, clone1A8, dilution 1:100)
16. anti-CD11c- BV605 (Biolegend, cat#117334, clone N418, dilution 1:100)
17. anti-SiglecF - BV750 (BD, cat# BD552125, clone E50-2440, dilution 1:100, lot: 1040516)
18. anti-MHC II – BV 785 (Biolegend, cat# 107645, clone M5/114.15.2, dilution 1:100, lot: B309531)
19. anti-AnnexinV - FITC (R&D Systems, cat# 4830-01-K, dilution 1:100, lot: P218017)
20. anti-F4/80- PE (Biolegend, cat# 123109, clone BM8, dilution 1:100, lot: B340064)
21. anti-SiglecF- PE (BD, cat#562068, clone E50-2440, dilution 1:100, lot: 7058859)
22. anti-CD4- PECy7 (Invitrogen, cat# 25-0041-82, clone GK1.5, dilution 1:100, lot: B297465)

Validation	All antibodies are commercially purchased from credible sources and have been validated by the manufacturer. All antibodies used have been cited several times by other investigators and were chosen using BenchSci, an AI software that identifies credible and reproducible reagents such as antibodies.<br><br>All antibodies from Biolegend have been validated for use in mouse samples and flow cytometry from the manufacturer.<br>The anti-p21 antibody (Santa Cruz, Cat# sc-6246, clone F-5, dilution 1:200, lot: L2921) was validated by Santa Cruz for western blot in HCT 116 and Hep G2 cell lysates. The anti-betaTubulin antibody (Cell Signaling Technology, Cat# 2146S, dilution 1:1000, lot: 9) and anti-GAPDH antibody (Cell Signaling Technology, cat# 2118s, clone 14C10, dilution 1:1000, lot: 14) were validated by the manufacturer in various cell lines including HeLA, C6, HUVEC, and ZF4. The anti-CD45 microbeads were validated by Miltenyi using mouse spleenocytes. The anti-CD31 microbeads were validated by Miltenyi using bEnd.3 endothelial cells. The anti-CD326 antibody (Miltenyi, Cat # 130-097-418, lot: 5200700545) was validated by Miltenyi.

Eukaryotic cell lines

Policy information about cell lines

Cell line source(s)	MH-S cells were obtained from ATCC
Authentication	MH-S cells were obtained from and authenticated by ATCC through ATCC's routine short tandem repeat profiling authentication procedures.
Mycoplasma contamination	MH-S cell obtained from ATCC tested negative for mycoplasma
Commonly misidentified lines<br>(See ICLAC register)	No commonly misidentified cell lines were used

Animals and other organisms

Policy information about studies involving animals; ARRIVE guidelines recommended for reporting animal research

Laboratory animals	Young (2-4 months) and aged (18-22 months) female C57BL/6N mice were obtained from Charles Rivers and the National Institute of Aging rodent facility at Charles Rivers. Male UM-HET3 mice were kindly gifted by Dr. Richard Miller at the University of Michigan. The male UM-HET3 mice were aged at the Glenn Center on Aging at the University of Michigan. MitoQC mice were donated from the lab of Dr. Ian Ganley at the University of Dundee and originally generated by Taconic Artemis 44. The MitoQC mice were then bred and housed within the animal facility at the North Campus Research Complex at the University of Michigan. Male and female EP2 KO (Ptger2 KO, Jackson Laboratory, strain #004376), on the C57BL/6 background, were obtained from the lab of Dr. Marc Peters-Golden at the University of Michigan. All mice were maintained on a 12-hour light-dark cycle with free access to food and water within a specific-pathogen-free facility. Mice were monitored for at least 1 week after arrival to our facilities for signs of stress and/or disease. All mouse housing rooms are maintained at 72 +/- 2 degrees Fahrenheit and 30-70% relative humidity.
Wild animals	No wild animals were used in this study
Field-collected samples	No field-collected samples were used in this study
Ethics oversight	The mouse work was performed under the study protocol PRO00008941, as approved by the University of Michigan Institutional Animal Care and Use Committee

Note that full information on the approval of the study protocol must also be provided in the manuscript.

Human research participants

Policy information about studies involving human research participants

Population characteristics	There were 22 human samples used. The mean (standard deviation) of the age of the donors is 43.7 (25.6). 54.5% of the participants were female.<br>Ethnicity, n (%): Caucasian, 16 (72.7%); African American, 3 (13.6%), Hispanic, 3 (13.6%)<br>Smoking status, n (%): non-smoker, 7 (31.8%); ex-smoker, 8 (36.3%); smoker, 7 (31.8%)
Recruitment	Lung samples that were not suitable for organ transplantation were donated from the Gift of Life
Ethics oversight	The use of human samples was approved by IRB at Temple University

Note that full information on the approval of the study protocol must also be provided in the manuscript.

Flow Cytometry

Plots

Confirm that:

☒ The axis labels state the marker and fluorochrome used (e.g. CD4-FITC).

☒ The axis scales are clearly visible. Include numbers along axes only for bottom left plot of group (a 'group' is an analysis of identical markers).

☒ All plots are contour plots with outliers or pseudocolor plots.

☒ A numerical value for number of cells or percentage (with statistics) is provided.

Methodology

Sample preparation	Cells were obtained from the BALF, single-cell suspension of lungs or cell culture. The BALF was collected by lavaging the lungs twice with 1mL of cold sterile PBS. Cells in the BALF were resuspended into FACS buffer (PBS + 2mM EDTA + 4% FBS) for flow cytometry staining. Lungs were digested with 1 mg/ml Collagenase D (Roche, COLLD-RO) and 10 U/ml DNase (Roche, 04536282001) for 45 minutes at room temperature with agitation. After digestion, the lungs were minced and passaged through a 100µm cell strainer. To remove red blood cells, cells were incubated with red blood cell lysis buffer (Biolegend, 420301) for 3 minutes. Cells were stained with a live/dead viability dye according to manufacturer's instructions (ThermoFisher, L34966). Next, Fc blocking was performed by incubation with anti-CD16/32 antibody for 20 minutes (Biolegend, 101320). Following, cells were stained with the desired surface markers and fixed in 4% paraformaldehyde for 25 minutes. Cells were washed twice and resuspended in FACS buffer until analysis.
Instrument	Flow cytometry was performed using the ZE5 Cell Analyzer (BioRad) of the Flow Cytometry Core at the University of Michigan
Software	The flow cytometry data was collected using the Everest Software (Biorad) and then analyzed using FlowJo (version 10.8.0)
Cell population abundance	Our method for isolating type II AECs typically yields approximately 85% purity of prosurfactant-C+ cells as measured by flow cytometry.
Gating strategy	Using the FSC/SSC gating, debris was removed by gating on the main cell population. Then singlets were gated on using a doublet-discriminator. The positivity threshold was determined based on the non-stained and the fluorescence-minus-one controls.

☒ Tick this box to confirm that a figure exemplifying the gating strategy is provided in the Supplementary Information.
